# A mixed-methods approach to understand university students’ perceived impact of returning to class during COVID-19 on their mental and general health

**DOI:** 10.1371/journal.pone.0279813

**Published:** 2023-01-03

**Authors:** Qinglan Ding, Mark Daniel Ward, Nancy Edwards, Emily Anna Wu, Susan Kersey, Marjorie Funk

**Affiliations:** 1 College of Health and Human Sciences, Purdue University, West Lafayette, IN, United States of America; 2 College of Science, Purdue University, West Lafayette, IN, United States of America; 3 School of Nursing, Purdue University, West Lafayette, IN, United States of America; 4 Krannert School of Management, Purdue University, West Lafayette, IN, United States of America; 5 School of Nursing, Yale University, West Haven, CT, United States of America; Newcastle University, UNITED KINGDOM

## Abstract

**Purpose:**

This study investigated the prevalence and risk factors of mental and general health symptoms among university students attending in-person and online classes during COVID-19. We also explored their experiences returning to in-person classes and their views on the university’s COVID-19-related policies.

**Methods:**

In this sequential explanatory mixed-methods study (2020–2021), U.S. university student respondents (N = 1030; 603 women [58.5%], 907 [88.1%] aged 18–24 years) completed a quantitative, cross-sectional survey assessing their mental and general health symptoms experienced while taking classes during the COVID-19 pandemic. The survey link was distributed through social media and email invitations. Three separate follow-up focus groups (n = 27), consisting of an average of nine focus group respondents who had completed the quantitative survey per group, were conducted using a semi-structured interview guide. Focus group respondents provided qualitative responses on their experiences returning to class during COVID-19 and adhering to COVID-19-related policies.

**Results:**

The prevalence of mental health symptoms among survey respondents were 57.6% (n = 593) for depression, 41.5% (n = 427) for anxiety, and 40.8% (n = 420) for stress. Over 90% of respondents reported perceptions of good general health. Female respondents and respondents identified as non-binary gender had an increased risk for mental health symptoms compared to male respondents. Respondents with preexisting medical conditions had an increased risk for worse general health. Themes identified through qualitative analysis included (1) attending class during COVID-19 is associated with unhealthy behaviors, and poor health, (2) perceived challenges of online learning and increased feelings of isolation, (3) demand for COVID-19 policy reform and greater transparency of COVID-19 statistics; (4) difficulties in adhering to COVID-19 policies; and (5) concerns about acquiring and transmitting COVID-19.

**Conclusions:**

Our findings indicate that university students attending classes during the pandemic are experiencing negative mental health impacts. Although students were aware of COVID-19-related policies, many found it challenging to comply. Broad acceptance of COVID-19 policies will require greater transparency and information sharing.

## Introduction

The coronavirus disease 2019 (COVID-19) continues to spread globally, with more than 628 million confirmed cases and 6,580,107 deaths in 188 countries as of October 25, 2022 [1]. Before COVID-19 vaccines became widely available, more than 168 million students worldwide were affected due to COVID-19 restrictions [2, 3]. Because of the lack of a standard template for determining whether to educate students remotely, bring them back into the classroom, or create a hybrid model combining both, some universities in the U.S. decided to resume teaching students full-time using a combination of in-person and remote education in the fall/autumn term of 2020 (August-December) [4]. Although a growing body of evidence shows that the COVID-19 pandemic and strict social distancing measures negatively affect young adults’ mental and physical health [1, 5], prolonged closures in universities have been associated with worsening mental health and learning outcomes in university students [3, 6]. Prior to COVID-19, the 2019 Annual Report of the Center for Collegiate Mental Health reported over 60% of students seeking counseling had anxiety disorders among 82,685 respondents from 188 universities across the U.S [7]. A recent interview survey conducted among 195 university students in the U.S. showed that nearly 71% of university students reported increased stress and anxiety due to the COVID-19 pandemic, compared to pre-COVID-19 [8]. Additionally, data from the same university found that nearly 50% of university students had a moderate-to-severe level of depression, and 38.5% had a moderate-to-severe level of anxiety [9].

The mental health of university students related to COVID-19 reported by previous studies is a matter of growing concern. Although universities across the globe are re-opening as we enter various recovery phases from COVID-19, few studies have used a qualitative or mixed-methods approach to understand students’ perceptions, attitudes, or beliefs concerning the mental and general health effects of attending class during COVID-19, and how university openings under different safety protocols further impact their mental health and general well-being.

The lack of qualitative aspects in understanding the individual effects of returning to class during COVID-19 leaves two important questions unanswered for policymakers and researchers: first, what are university students’ on-campus and online learning experiences related to the COVID-19 pandemic? Second, how can learners be supported to optimize coping strategies to mitigate mental health symptoms and facilitate the implementation of preventive interventions in the future? Although a rise in psychological symptoms of anxiety, depression, and coping response to stress are expected during extraordinary circumstances, heightened levels of psychological distress can negatively impact academic performance for learners such as university students [10]. University safety protocols designed to reduce COVID-19 transmission rates may also inevitably affect students’ mental health and general well-being. Despite this, there is little knowledge regarding university students’ compliance with safety protocols designed to keep the pandemic controlled while returning to campus during COVID-19.

To understand university students’ experiences of attending class in-person or remotely during COVID-19, we designed and conducted a study in a U.S.-based university that remained open, with more than 40,000 students taking courses on campus during the fall/autumn term of 2020. Using social media as a recruitment platform and a sequential mixed-methods approach, we aim to elicit perceptions, attitudes, and experiences from university students regarding the health impacts of attending school during COVID-19. The quantitative analyses investigated the prevalence of symptoms of depression, anxiety, stress, and poor general health, and identified associated risk factors. The qualitative data are drawn from a subsample of university students who had completed the quantitative survey, with a focus on examining how students interpret and give meaning to their class participation experiences, exploring their perceptions and beliefs regarding barriers to compliance with COVID-19 related safety protocols, and ways they can be supported to optimize coping strategies to mitigate mental and general health symptoms and facilitate the implementation of preventive interventions in the future.

## Methods

### Study design

We used a mixed-methods sequential explanatory design consisting of quantitative data and analysis (anonymous online survey) in phase 1, followed by qualitative data collection and analysis (focus group discussions) in phase 2 to enhance understanding of the quantitative results [10].

We also gave slightly greater weight to qualitative data and used the quantitative findings to inform purposeful sampling, refine focus group questions, and analytic paths in the qualitative phase. The quantitative and qualitative phases were connected when selecting respondents for qualitative study and developing interview protocol based on quantitative analysis results. The results of the quantitative and qualitative phases were integrated during the discussion of the findings of the entire study (S1 Fig). We anticipated that the combination of quantitative and qualitative studies would provide a rich and full exploration of factors contributing to worsening mental and general health symptoms among university students attending class during COVID-19 and serve to guide the development of support strategies with consideration of these significant factors.

### Target population

The study’s target population was university students attending class partially or completely in-person at a public university in the Midwestern U.S. in the fall/autumn term of 2020. Students who met the survey inclusion criteria (N = 6,000) were invited to participate, and their status varied in degree progress, undergraduate/graduate levels, and majors/fields of study pursued. The study sample size was calculated based on one of our primary outcomes, DASS-21 depression scale, as it is known as the most common and significant public health concern with serious clinical impact compared to anxiety [11]. Based on previous research, we expected university students to experience a greater prevalence of depressive symptoms than similarly-aged adults in the general population [12, 13]. We used G*Power 3.1.9.6 [14]. software for the sample size calculation, specifying a two-tailed test, α = 0.05; β = 0.05 (95% power); a constant proportion of depressive symptoms in youth in the general population during the COVID-19 pandemic = 0.252 [15], an alternative proportion through Naïve guess according to Cohen (0.5 = medium effect size) [16], and an attrition rate of 20%. The required minimum sample size based on the priori power analysis was estimated at 62 subjects.

### Quantitative phase

#### Survey data collection

We used a cross-sectional web-based survey design in the first phase to collect information from student respondents, including demographics, mental and general health symptoms. At the end of the survey, respondents were given the option to include their email for focus group involvement. The survey was administered via Qualtrics (Provo, UT) during October 2020-December 2020 and was accessed through the URL link. Study recruitment was conducted mainly by convenience sampling. Using Reddit, Facebook, WhatsApp, and Instagram as recruitment platforms, a social media advertising campaign was designed to disseminate the survey link (S1 Method). The survey link was also distributed through an email campaign that included a random sample of 5,000 students enrolled in the 2020 fall/autumn semester. The survey was designed to take approximately 15 minutes to complete.

#### Survey measurements

The quantitative survey contains three parts. The first part gathered demographic information, including age, gender, race, major/fields of study, education level, type of residence, attend in-person class or not, and pre-existing medical conditions. The second part of the survey consisted of 2 standardized and validated questionnaires, including the Depression, Anxiety, and Stress Scale (DASS) and the EuroQol-5D (EQ-5D) questionnaire [17, 18]. Both EQ-5D and DASS have demonstrated feasibility, reliability, and validity in young adults [19]. Respondents were asked to complete the survey questions based on their experiences attending class in the 2020 fall academic term. The last part of the survey assessed respondents’ willingness to participate in the focus group discussions and their preferred way of being contacted if they were selected to participate.

*DASS*. We assessed students’ mental health using DASS [17]. DASS is a widely used, validated 21-item self-administered instrument designed to determine the severity of depression, anxiety, and stress symptoms in the past month, with higher scores on each DASS scale indicating more severe levels of depression and anxiety stress [17]. The DASS is not intended to diagnose mental disorders but measures the prevalence of depression, anxiety, and stress symptoms and severity over the prior week [11, 20]. DASS-21 scores were used to evaluate the severity of depression, anxiety, and stress symptoms in this study (mild: 10–13, 8–9, 15–18, respectively; moderate: 14–20, 10–14, 19–25, respectively; and severe to extremely severe: ≥21, ≥15, ≥26, respectively).

*EQ-5D*. We assessed students’ general health status using the EQ-5D scale, a generic measure of health status. The EQ-5D uses a descriptive system of 5-dimensions (mobility, self-care, usual activities, pain/discomfort, and anxiety/depression) [18, 21]. The EQ-5D has five levels for each dimension (no problems = 1, slight problems = 2, moderate problems = 3, severe problems = 4, extreme problems = 5) [18, 21]. For our analysis, EQ-5D scores in each of the five dimensions were aggregated into three categories (no problem, slight problems, and moderate-to-severe problems). EQ-5D also included a visual analog scale (VAS) with a grade ranging from 0 (worst possible health status) to 100 (best imaginable health state) [22].

### Quantitative data analysis

Descriptive statistics were used to present respondents’ demographic characteristics. Prevalence of mental and general health symptoms was calculated using the cutoff scores mentioned above and reported as percentages of cases in each subgroup. Chi-square or Fisher Exact tests were used to compare the prevalence of mild-to-extremely severe symptoms in different subgroups. To determine demographic characteristics associated with moderate-to-extremely severe mental health and general health symptoms, unadjusted and adjusted logistic regression analyses were performed, and odds ratios (ORs) and 95% confidence intervals (CIs) were presented. For overall health (numeric variable), general linear models were used instead. Two-sided Wald tests were conducted to determine where the ORs in regression models were statistically significant. A sensitivity analysis was undertaken, including only respondents who attended in-person classes, to ensure that the findings of the main analysis were robust. For all analyses, the significance level was set to P < .05. All statistical analyses were performed using SAS (9.4).

### Qualitative phase

#### Focus group participants’ selection

We used semi-structured interviews and focus group discussions to collect data in the second (qualitative) phase. Two hundred and seventy eligible respondents who completed the online survey also expressed interest in participating in the focus group discussion. Among the 270 prospective focus group participants, we used purposive sampling to select 1–2 participants that minimally to maximally vary on 4 factors (majors/fields of study, gender, races/ethnicities, had chronic medical conditions or not) [23]. The 4 factors were selected based on literature review and survey findings from the quantitative data. This procedure yielded 36 participants and 27 of them consented to participate.

#### Focus group procedures

Three focus groups (9 participants per group) were held virtually between November 25-December 15, 2020, utilizing videoconferencing software Zoom (San Jose, CA) after the completion of quantitative data collection and analyses. Focus groups were semi-structured, lasting approximately 90 minutes each and facilitated by three psychiatric/mental health nurse practitioners that were not involved in the design or analysis of the study. The semi-structured interview guide was developed based on the quantitative results from the first phase of the study and the aims of gathering participants’ views of the university’s on-campus COVID-19 safety protocols and their experiences of adherence to these protocols. To gain a deeper understanding of our quantitative findings, we also explored why certain factors (such as presence of medical conditions) contributed to their mental and general health while attending class during COVID-19.

The full semi-structured focus groups guide is included in S2 Method. In brief, each of the three focus groups were divided into three parts, the first part focused on discussing the impact of attending class or events on campus during the pandemic on participants’ mental and general well-being. The second part discussed the participants’ feelings and experiences in complying with COVID-19-related safety protocols while attending classes on campus. The third part of each focus group explicitly elaborates participants’ views regarding the COVID-19-related policies and safety protocols as well as the university’s communication around COVID-19-related policies.

#### Data analysis

Each focus group was audiotaped, transcribed, and imported into NVivo Pro (12.3) for analysis. Iterative inductive thematic analysis was used to sift, chart, and sort transcripts in accordance with key issues and themes following five steps: familiarization, identification of a thematic framework, creation of indexing, creation of charting and mapping, and interpretation [24, 25]. Two researchers (QD and EW) analyzed the transcripts independently at each stage of the process, took notes, and discussed similarities and differences before the next iteration. The development of the thematic map and findings interpretation were discussed and refined during bi-weekly meetings of the research team. Finally, common themes were integrated, and findings were compiled. Saturation was reached after the third focus group, as thematic analysis showed that no new codes, categories, or themes emerged from the qualitative data. Detailed information about the qualitative analysis is described in S3 Method and S1 Fig.

#### Ethical considerations

To protect our respondents’ confidentiality, the survey was anonymous, and respondents in the first phase were assigned a random ID number in the quantitative analyses; the respondents selected for focus groups were given unique passwords to access the virtual focus groups. We also removed names and gender-related pronouns from quotations selected for illustrations. Study recruitment and procedures were approved by the Institutional Review Board of Purdue University. Written informed consent was received online before respondents answered survey questions, and verbal consent was obtained before the focus group discussions.

## Results

### Characteristics of respondents

Of 1047 students that opened the survey link, 1042 student respondents provided consent to participate in the survey (S2 Fig). Twelve respondents who did not complete the survey or give valid demographic information were excluded from the quantitative analysis. The 1030 respondents (N = 1030) included in the final survey analysis represented undergraduate and graduate students from 195 majors across 11 departments/colleges (Table 1). The overall response rate was 98.38% (1030 respondents out of 1047), and the sampling fraction was 0.13 (6000 of 45,869 total enrolled students in the fall/autumn 2020 semester). Of the total sample, 603 respondents (58.54%) were women, 15 (1.46%) identified as a gender other than male or female, and 907 respondents (88.06%) were aged 18–24 years. Of the 1030 survey respondents, 714 (69.32%) were non-Hispanic whites, 212 (20.58%) were Asian, and 11 (1.07%) were Black/African American. The survey included data from 865 (83.98%) undergraduate student respondents, 807 (78.35%) of the survey respondents were taking at least one in-person class in the 2020 fall/autumn term, and 474 (46.0%) were living in an on-campus university residence. Our survey also included responses from 202 respondents (19.61%) with pre-existing medical conditions. Demographics information of our focus group participants is listed in S2 Table.

**Table 1:** Demographics of overall population in quantitative study.

Characteristic		Total (N = 1030)
**Age range, n (%)**		
	15–17	9 (0.87)
	18–24	907 (88.06)
	25–29	75 (7.28)
	30–35	28 (2.72)
	>35	6 (0.58)
**Race, n (%)** (n (%) may not equal total for separate gender categories due to missing values.)		
	White	714 (69.32)
	Asian	212 (20.58)
	Black	11 (1.07)
	More than one race or other	81 (7.86)
**Level of education, n (%)**		
	Undergraduate	865 (83.98)
	Graduate	165 (16.02)
**Has in-person classes for Fall 2020? n (%)**		
	Yes	807 (78.35)
	No	223 (21.65)
**Living arrangement, n (%)**		
	University residences	474 (46.02)
	Off-campus housing	446 (43.30)
	Staying at home	59 (5.73)
	Currently outside of the United States	51 (4.95)
**Has medical conditions? n (%)**		
	No	828 (80.39)
	Yes	202 (19.61)
**Work, n (%)**		
	Unemployed	617 (59.90)
	Part-time	358 (34.76)
	Full-time	55 (5.34)
**How did you hear about our study? n (%)**		
	Electronic mailing list	746 (72.43)
	Social networking platform	208 (20.19)
	Word of mouth (ex.: peers, friends)	70 (6.80)
	Other	33 (3.20)

### Prevalence of symptoms of depression, anxiety, and stress

The prevalence of moderate-to-extremely severe DASS-21 symptoms among the student respondents was 57.6% (593/1030) for depression, 41.5% (427/1030) for anxiety, and 40.8% (420/1030) for stress (Table 2). The prevalence of moderate-to-extremely severe anxiety/depression symptoms on the EQ-5D subscale was 60.1% (619/1030) (Table 3). The prevalence of moderate-to-severe mental health symptoms was high among females (DASS-D, 62.5%; DASS-A, 47.9%; DASS-S, 46.8%; EQ-5D anxiety/depression, 65.8%), respondents of other genders (DASS-D, 80.0%; DASS-A, 66.6%; DASS-S, 60.0%; EQ-5D anxiety/depression, 73.3%) and those with pre-existing medical conditions (DASS-D, 67.3%; DASS-A, 58.4%; DASS-S, 55.5%; EQ-5D anxiety/depression, 74.8%). The prevalence of anxiety or depression symptoms was also high among non-Hispanic whites (DASS-D, 60.5%; EQ-5D anxiety/depression, 65.5%), respondents from the sciences/health sciences departments (EQ-5D anxiety/depression, 65.2%), and those who were working full- or part-time (EQ-5D anxiety/depression, 65.4%). Symptom profiles of focus group respondents are presented in S3.1 and S3.2. Table.

**Table 2:** Prevalence of symptoms of depression, anxiety, and stress (DASS-21) stratified by survey respondent characteristics.

		Depression				Anxiety				Stress			
Characteristic		Respondents with symptom, No. (%)			P value	Respondents with symptom, No. (%)			P value	Respondents with symptom, No. (%)			P value
		Mild	Moderate to Extremely Severe	Total, No. (%) [95% CI]		Mild	Moderate to Extremely Severe	Total, No. (%) [95% CI]		Mild	Moderate to extremely severe	Total, No. (%) [95% CI]	
**Overall**		141 (13.7)	593 (57.6)	734 (71.3) [68.5–74.0]		76 (7.4)	427 (41.5)	503 (48.9) [45.8–51.9]		109 (10.6)	420 (40.8)	529 (51.4) [48.3–54.4]	
**Gender**					0.369				0.058				0.377
	Male	55 (13.3)	204 (49.6)	259 (62.9) [58.2–67.5]		32 (7.7)	128 (31.1)	160 (38.8) [34.1–43.5]		40 (9.7)	129 (31.3)	169 (41.0) [36.3–45.8]	
	Female	85 (14.1)	377 (62.5)	462 (76.6) [73.2–80.0]		44 (7.3)	289 (47.9)	333 (55.2) [51.3–59.2]		68 (11.3)	282 (46.8)	350 (58.0) [54.1–61.9]	
	Other	1 (6.7)	12 (80.0)	13 (86.7) [69.5–100]		0	10 (66.7)	10 (66.7) [42.8–90.5]		1 (6.7)	9 (60.0)	10 (66.7) [42.8–90.5]	
**Race**					0.258				**0.016**				0.221
	White	96 (13.4)	432 (60.5)	528 (73.9) [70.7–77.2]		47 (6.6)	321 (44.9)	368 (51.5) [47.9–55.2]		76 (10.6)	317 (44.4)	393 (55.0) [51.4–58.7]	
	Non-white	45 (14.2)	161 (50.9)	206 (65.2) [60.0–70.4]		29 (9.2)	106 (33.5)	135 (42.7) [37.3–48.2]		33 (10.4)	103 (32.6)	136 (43.0) [37.6–48.5]	
**Age range**					0.527				0.482				0.773
	15–24	129 (14.1)	532 (58.1)	661 (72.2) [69.3–75.1]		70 (7.6)	382 (41.7)	452 (49.3) [46.1–52.6]		96 (10.5)	374 (40.8)	470 (51.3) [48.1–54.6]	
	≥ 25	12 (10.5)	61 (53.5)	73 (64.0) [55.2–72.8]		6 (5.3)	45 (39.5)	51 (44.7) [35.6–53.9]		13 (11.4)	46 (40.4)	59 (51.8) [42.6–60.9]	
**Education**					0.776				0.446				0.876
	Under-graduate	118 (13.6)	502 (58.0)	620 (71.7) [68.7–74.7]		62 (7.2)	363 (41.9)	425 (49.1) [45.8–52.5]		91 (10.5)	348 (40.2)	439 (50.8) [47.4–54.1]	
	Graduate	23 (13.9)	91 (55.2)	114 (69.1) [62.0–76.1]		14 (8.5)	64 (38.8)	78 (42.3) [39.7–54.9]		18 (10.9)	72 (43.6)	90 (54.6) [46.9–62.1]	
**Living arrangement**					0.649				0.429				**0.021**
	Living in UR (UR: university residences)	66 (13.9)	265 (55.9)	331 (69.8) [65.7–73.9]		37 (7.8)	187 (39.5)	224 (47.3) [42.8–51.8]		57 (12.0)	168 (35.4)	225 (47.5) [42.9–51.9]	
	Not living in UR	75 (13.5)	328 (59.0)	403 (72.5) [68.8–76.2]		39 (7.0)	240 (43.2)	279 (50.2) [46.0–54.3]		52 (9.4)	252 (45.3)	304 (54.7) [50.5–58.8]	
**Work status**					0.322				0.549				0.062
	Employed	53 (12.8)	250 (60.5)	303 (73.4) [69.1–77.6]		31 (7.5)	190 (46.0)	221 (53.5) [48.7–58.3]		39 (9.4)	192 (46.5)	231 (55.9) [51.1–60.7]	
	Unemployed	88 (14.3)	343 (55.6)	431 (69.9) [66.2–73.5]		45 (7.3)	237 (38.4)	282 (45.7) [41.8–49.6]		70 (11.3)	228 (36.9)	298 (48.3) [44.4–52.2]	
Has in-person classes for Fall 2020? (Y/N) (Y/N: yes/no)					0.748				0.491				0.453
	Yes	111 (13.8)	474 (58.7)	585 (72.5) [69.4–75.6]		59 (7.3)	346 (42.9)	405 (50.2) [46.7–53.6]		87 (10.8)	321 (39.8)	408 (50.6) [47.1–54.0]	
	No	30 (13.5)	119 (53.4)	149 (66.8) [60.6–73.0]		17 (7.6)	81 (36.3)	98 (43.9) [37.4–50.5]		22 (9.9)	99 (44.4)	121 (54.3) [47.7–60.8]	
**Has medical conditions? (Y/N)**					0.057				**0.016**				0.235
	Yes	22 (10.9)	136 (67.3)	158 (78.2) [72.5–83.9]		11 (5.4)	118 (58.5)	129 (63.9) [57.2–70.5]		23 (11.4)	112 (55.4)	135 (66.8) [60.3–73.3]	
	No	119 (14.4)	457 (55.2)	576 (69.6) [66.4–72.7]		65 (7.9)	309 (37.3)	374 (45.2) [41.8–48.6]		86 (10.4)	308 (37.2)	394 (47.6) [44.2–50.9]	

**Table 3:** Severity of mobility, self-care, usual activities, pain/discomfort, and anxiety/depression symptoms (EQ-5D) stratified by survey respondent characteristics.

		Mobility				Self-care				Usual activities			
Characteristics		Respondents with difficulty, No. (%)				Respondents with difficulty, No. (%)				Respondents with difficulty, No. (%)			
		Slightly difficult	Moderately to extremely difficult	Total No. (%) [95% CI]	P value	Slightly difficult	Moderately to extremely difficult	Total No. (%) [95% CI]	P value	Slightly difficult	Moderately to extremely severe, n (%)	Total No. (%) [95% CI]	P value
**Overall**		59 (5.7)	13 (1.3)	72 (7.0) [5.4–8.6]		43 (4.2)	22 (2.1)	65 (6.3) [4.8–7.8]		212 (20.6)	240 (23.3)	452 (43.9) [40.9–46.9]	
**Gender**					0.269				0.078				0.761
	Male	21 (5.1)	2 (0.5)	23 (5.6) [3.4–7.8]		6 (1.5)	4 (1.0)	10 (2.5) [0.9–3.9]		77 (18.7)	84 (20.4)	162 (39.1) [34.4–43.8]	
	Female	34 (5.6)	11 (1.8)	45 (7.4) [5.4–9.6]		34 (5.6)	18 (3.0)	52 (8.6) [6.4–10.9]		131 (21.7)	149 (24.7)	280 (46.4) [42.5–50.4]	
	Other	4 (26.7)	0 (0.0)	4 (26.7) [4.3–49.1]		3 (20.0)	0 (0.0)	3 (20.0) [0–40.2]		4 (26.7)	7 (46.6)	11 (73.3) [50.9–95.7]	
**Race**					0.719				0.389				0.148
	White	44 (6.2)	11 (1.5)	55 (7.7) [5.8–9.7]		31 (4.3)	18 (2.5)	49 (6.8) [5.0–8.7]		147 (20.6)	181 (25.4)	328 (46.0) [42.3–49.6]	
	Non-white	15 (4.8)	2 (0.6)	17 (5.4) [2.9–7.9]		12 (3.8)	4 (1.3)	16 (5.1) [2.7–7.5]		65 (20.6)	59 (18.7)	125 (39.3) [33.9–44.6]	
**Age range**					0.349				0.599				0.114
	15–24	53 (5.8)	10 (1.1)	63 (6.9) [5.2–8.5]		41 (4.5)	20 (2.2)	61 (6.7) [5.0–8.3]		198 (21.6)	214 (23.4)	412 (45.0) [41.8–48.2]	
	≥ 25	6 (5.3)	3 (2.6)	9 (7.9) [2.9–12.8]		2 (1.8)	2 (1.8)	4 (3.6) [0.1–6.9]		14 (12.3)	26 (22.8)	41 (35.1) [26.3–43.9]	
**Education**					0.373				1.000				0.081
	Undergraduate	52 (6.0)	10 (1.2)	62 (7.2) [5.5–8.9]		38 (4.4)	19 (2.2)	57 (6.6) [4.9–8.2]		188 (21.7)	199 (23.0)	387 (44.7) [41.4–48.1]	
	Graduate	7 (4.3)	3 (1.8)	10 (6.1) [2.4–9.7]		5 (3.0)	3 (1.8)	8 (4.8) [1.6–8.1]		24 (14.6)	41 (24.9)	66 (39.4) [31.9–46.9]	
**Living arrangement**					0.084				0.663				0.078
	Living in UR (UR: university residences)	38 (8.0)	5 (1.1)	43 (9.1) [6.5–11.7]		22 (4.6)	10 (2.2)	32 (6.8) [4.5–9.0]		105 (22.1)	99 (20.9)	204 (43.0) [38.6–47.5]	
	Not living in UR	21 (3.8)	8 (1.4)	29 (5.2) [3.4–7.1]		21 (3.8)	12 (2.1)	33 (5.9) [4.0–7.9]		107 (19.2)	141 (25.4)	249 (44.6) [40.5–48.7]	
**Work status**					0.101				0.191				**0.036**
	Employed	22 (5.3)	8 (1.9)	30 (7.3) [4.8–9.8]		23 (5.6)	8 (1.9)	31 (7.5) [4.9–10.5]		80 (19.4)	114 (27.6)	195 (47.0) [42.2–51.8]	
	Unemployed	37 (6.0)	5 (0.8)	42 (6.8) [4.8–8.8]		20 (3.2)	14 (2.3)	34 (5.5) [3.7–7.3]		132 (21.4)	126 (20.4)	258 (41.8) [37.9–45.7]	
Has in-person classes for Fall 2020? (Y/N) (Y/N: yes/no)					0.708				0.655				0.588
	Yes	48 (5.9)	10 (1.2)	58 (7.2) [5.4–8.9]		38 (4.7)	21 (2.6)	59 (7.3) [5.5–9.1]		173 (21.4)	191 (23.7)	364 (45.1) [41.7–48.5]	
	No	11 (4.9)	3 (1.4)	14 (6.3) [3.1–9.5]		5 (2.2)	1 (0.5)	6 (2.7) [0.5–4.8]		39 (17.5)	49 (22.0)	88 (39.5) [33.1–45.9]	
**Has medical conditions? (Y/N)**					0.556				0.078				0.042
	Yes	28 (13.9)	5 (2.5)	33 (16.4) [11.2–21.4]		12 (5.9)	11 (5.5)	23 (11.4) [7.0–15.8]		45 (22.2)	71 (35.2)	116 (57.4) [50.6–64.2]	
	No	31 (3.7)	8 (1.0)	39 (4.7) [3.3–6.2]		31 (3.7)	11 (1.3)	42 (5.0) [35.8–65.7]		167 (20.2)	169 (20.4)	336 (40.6) [37.2–43.9]	
				Pain/discomfort				Anxiety/depression					
**Characteristics**				Respondents, No. (%)				Respondents, No. (%)				
				Slightly difficult	Moderately to extremely difficult	Total No. (%) [95% CI]	P	Slightly difficult	Moderately to extremely difficult		Total No. (%) [95% CI]	P	
**Overall**				256 (24.9)	92 (8.9)	348 (33.8) [30.9–36.7]		261 (25.3)	616 (59.8)		877 (85.2) [82.9–87.3]		
**Gender**							0.778					**0.025**	
	Male			94 (22.8)	30 (7.3)	124 (30.1) [25.7–34.5]		114 (27.7)	210 (50.9)		324 (78.6) [74.7–82.6]		
	Female			157 (26.0)	60 (10.0)	217 (36.0) [32.2–39.8]		144 (23.9)	395 (65.5)		539 (89.4) [86.9–91.8]		
	Other			5 (33.3)	2 (13.4)	7 (46.7) [21.4–71.9]		3 (20.0)	11 (73.3)		14 (93.3) [80.7–100]		
**Race**							0.963					**0.012**	
	White			183 (25.6)	66 (9.3)	249 (34.9) [31.4–38.4]		176 (24.7)	466 (65.3)		642 (90.2) [87.7–92.1]		
	Non-white			73 (23.1)	26 (8.2)	99 (31.3) [26.2–36.4]		85 (26.9)	150 (47.5)		235 (74.7) [69.5–79.2]		
**Age range**							0.279					0.517	
	15–24			228 (24.9)	78 (8.5)	306 (33.4) [30.4–36.5]		230 (25.1)	552 (60.3)		782 (85.4) [83.1–87.7]		
	≥ 25			28 (24.6)	14 (12.3)	42 (36.9) [27.9–45.7]		31 (27.2)	64 (56.1)		95 (83.3) [76.5–90.2]		
**Education level**							0.423					0.342	
	Undergraduate			215 (24.9)	74 (8.5)	289 (33.4) [30.3–36.6]		214 (24.7)	521 (60.2)		735 (84.9) [82.6–87.4]		
	Graduate			41 (24.9)	18 (10.9)	59 (35.8) [28.4–43.1]		47 (28.5)	95 (57.6)		142 (86.1) [80.8–91.4]		
**Living arrangement**							0.656					0.649	
	Living in UR (UR: university residences)			121 (25.5)	41 (8.7)	162 (34.2) [29.9–38.5]		123 (25.9)	280 (59.1)		403 (85.0) [81.8–88.2]		
	Not living in UR			135 (24.3)	51 (9.2)	186 (33.5) [29.5–37.4]		138 (24.8)	336 (60.4)		474 (85.2) [82.3–88.2]		
**Work status**							0.332					**0.039**	
	Employed			102 (24.7)	42 (10.2)	144 (34.9) [30.3–39.5]		94 (22.8)	268 (64.9)		362 (87.7) [84.5–90.8]		
	Unemployed			154 (25.0)	50 (8.1)	204 (33.1) [29.4–36.8]		167 (27.1)	348 (56.4)		515 (83.5) [80.5–86.4]		
**Has in-person classes for Fall 2020? (Y/N) (Y/N: yes/no)**							0.637					0.451	
	Yes			197 (24.4)	73 (9.1)	270 (33.5) [30.2–26.7]		203 (25.2)	493 (61.1)		696 (86.3) [83.9–88.6]		
	No			59 (26.5)	19 (8.5)	78 (35.0) [28.7–41.2]		58 (26.0)	123 (55.2)		181 (81.2) [76.0–86.3]		
**Has medical conditions? (Y/N)**							**< .001**					**< .001**	
	Yes			56 (27.7)	44 (21.8)	100 (49.5) [42.6–56.4]		36 (17.8)	150 (74.3)		186 (92.1) [88.4–95.8]		
	No			200 (24.2)	48 (5.8)	248 (30.0) [26.8–33.1]		225 (27.2)	466 (56.3)		616 (83.5) [80.9–85.9]		

### Prevalence of general health problems

Table 3 shows the prevalence of general health problems (mobility, self-care, usual activities, and pain/discomfort) reported on the EQ-5D profile by respondents’ characteristics.

The EQ-5D VAS median score (interquartile range) for respondents was 75 (60–85) (S4 Table). Most respondents reported perceptions of good general health while attending class during COVID-19. The proportion of respondents who reported moderate-to-severe problems with usual activities (e.g., study, housework, leisure activities) was higher in those with medical conditions than those who did not (35.1% vs. 20.4%).

### Demographic characteristics associated with mental and general health symptoms

In the multivariable analysis, respondents who identified as other genders had nearly four times the risk for mental health symptoms compared with male respondents (adjusted ORs: 11.53 [95% CI 1.46–90.96] for DASS-depression, 5.13 [95% CI 1.51–17.38] for DASS-anxiety, and 3.93 [95% CI 1.26–13.62] for DASS stress) (S5 Table). Compared with male respondents, female respondents were also susceptible to symptoms of depression (adjusted ORs: 1.56; 95% CI 1.18–2.05), anxiety (adjusted ORs: 1.82; 95% CI 1.37–2.42), and stress (adjusted ORs: 1.80; 95% CI 1.36–2.38). Respondents who have pre-existing medical conditions (vs. those without) also had an increased risk of mental health symptoms (adjusted ORs: 1.59 [95% CI 1.42–2.26] for DASS-depression, 2.15 [95% CI 1.55–3.01] for DASS-anxiety, and 2.06 [95% CI 1.46–2.91] for DASS stress). Nonetheless, the non-white race was associated with a lower risk of having symptoms of depression (adjusted ORs, 0.74; 95% CI 0.55–0.99), anxiety (adjusted ORs, 0.71; 95% CI 0.52–0.95), and stress (adjusted ORs, 0.58; 95% CI 0.42–0.78). Unadjusted analyses for DASS-21 are shown in S6 Table.

Associations were also identified between respondents having pre-existing medical conditions and three general health symptoms, including difficulty in self-care, problems in usual activities, and pain and discomfort. Detailed results of the unadjusted and adjusted multivariable analyses for EQ-5D and EQ-5D-VAS are shown in S7–S9 Tables.

Sensitivity analyses assessing demographic characteristics associated with mental health (DASS-21) and general health (EQ-5D) restricted to respondents attending in-person classes only showed similar findings (S10.1–S10.3 Table).

### Themes from focus group discussions

**Fig 1:**
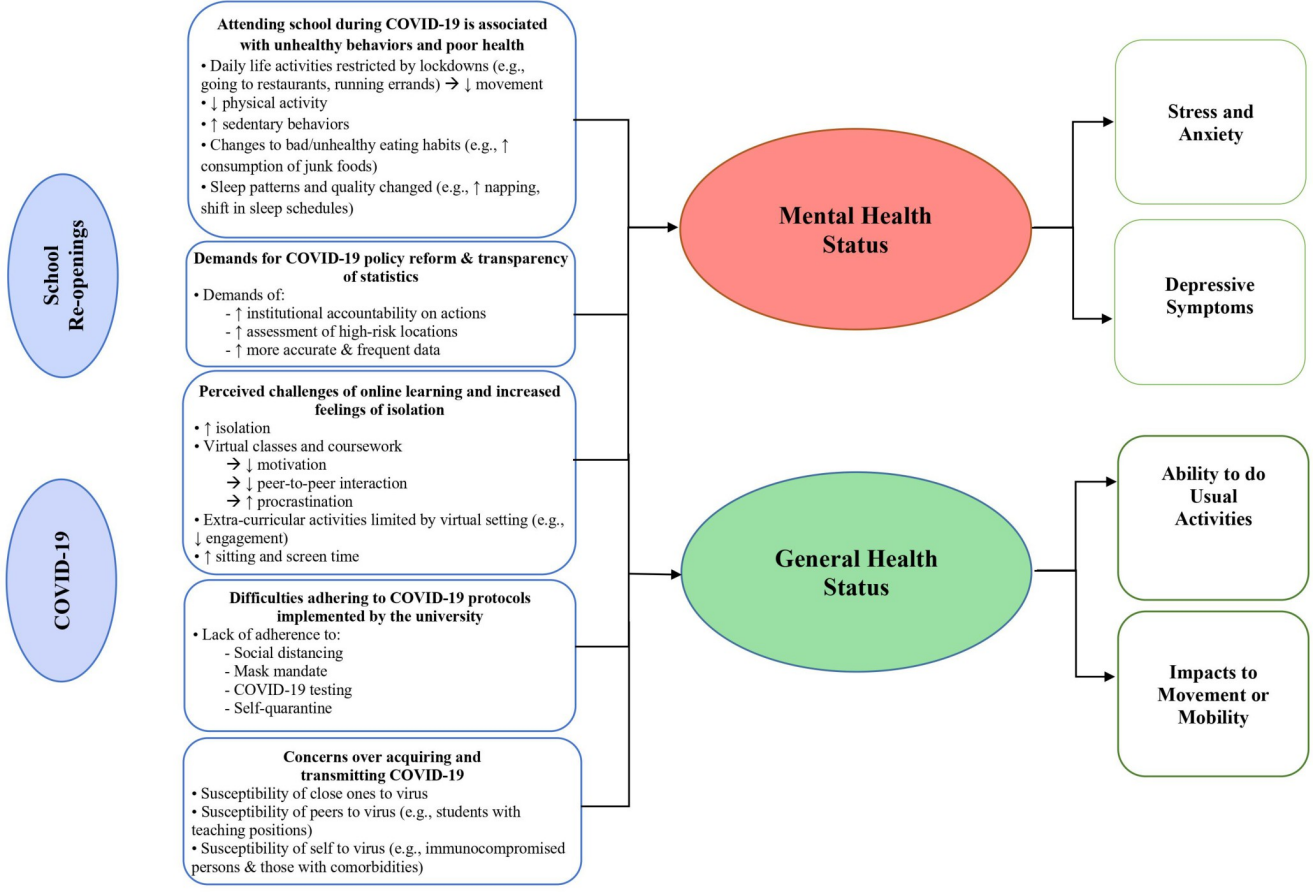
Themes observed from focus group study. The impact of attending university during COVID-19 on student respondents’ mental and general health status.

We identified five themes related to young adults’ views about the health impacts of attending class during the pandemic, their acceptance of COVID-19-related policy measures, and their experiences of adhering to these policies (Fig 1). Key themes and quotations from focus groups were presented in S11 Table. We discuss each of these themes along with related subthemes below.

#### Attending class during COVID-19 is associated with unhealthy behaviors and poor health

Many focus group respondents felt that attending class during COVID-19 significantly impacted their mental and general well-being (Fig 1). Internal and external factors that appeared to influence health negatively included increased sedentary behavior, lack of motivation for class, poor time management, and difficulty balancing class and work duties (e.g., “I definitely feel like I’m exercising a lot less since I’m not going to campus, I’m not walking between buildings. I don’t really have a reason to go outside since everything can be done indoors and the gym so far… it just makes it really difficult to motivate when you don’t have to move.") Not surprisingly, young adults with pre-existing medical conditions expressed more difficulty in maintaining a healthy lifestyle at the time (e.g., “I have joint issues already, which have been increasing lately from being sedentary, and I don’t want to.”).

#### Perceived challenges of online learning and increased feelings of isolation

Participants expressed how the shift to hybrid or online learning created challenges to learn and communicate with peers and instructors. Decreased peer interaction and motivation and increased procrastination were factors that exacerbated stress and depressive symptoms and impacted participants’ ability to perform daily activities (Fig 1). Some perceived that online learning exacerbated problems in academic settings, such as the inability to conduct routine “classroom conversation” with classmates and difficulties reaching out to professors. Many reported increased feelings of isolation from attending class online instead of in-person (e.g., “I’ve realized all my interactions with my colleagues like in the program where I teach are gone, and um then what that ends up doing psychologically, all the small issues with students or just the difficulties that are kind of endemic to teaching in graduate school, like I don’t have that camaraderie and that social support and routine that usually helps me take care of those things really quickly.”).

#### Demands for COVID-19 policy reform and greater transparency of university COVID-19 statistics

Many participants felt a need for changes in university COVID-19-related policies and greater transparency of COVID-19 statistics (e.g., “Within classrooms, and within other enclosed spaces… they’re not doing a really good job of informing people of what risks they might be under, who they might be in contact with, so contact tracing has also been very uninformative and um, not really doing a lot.”) Some participants expressed the need for more accountability among institutions rather than individual actions to prevent COVID-19 outbreaks. (e.g., “…we all wore our masks and came back to campus…but that doesn’t take into account different levels of spread or dangers.”). Others suggested that the university can do more to enhance protection and prevent COVID-19 transmission, such as identifying and managing high-risk locations and more transparency in campus COVID-19 statistics reporting. A few participants expressed concerns for personal safety and wellbeing due to a lack of COVID-19 risk management in large spaces, such as classes (Fig 1 and S11 Table). In contrast, a few participants spoke highly of the effort and commitment the institution had put forth during the pandemic (e.g., “Our school is doing a really good job compared to other universities and other states, and I think that, no matter where you are there are always going to be those unique instances of people refusing to follow the rules. But overall, we’ve been here on campus since, you know, the middle of August. And with as many cases as there have been, there have been only 6 hospitalizations and 0 deaths of those tested on campus… those are really good statistics compared to the rest of the world…”).

#### Difficulties in adhering to COVID-19-related policies and protocols implemented by the university

Despite awareness of COVID-19-related policies designed to protect them, some participants reported difficulties in adhering to these policies and protocols implemented by the university. Some also indicated feelings of uneasiness produced by their peers’ lack of adherence to COVID-19 policies and testing (e.g., “Someone I was standing with mentioned that their roommate had recently tested positive for COVID. And it’s like okay, I think it’s fairly reasonable to say if your roommate- who you’ve been in close contact with for a significant period of time, most likely without a mask on- has tested positive, you probably shouldn’t be out in public, even if you are still socially distanced, and what not.”) (Fig 1 and S11 Table). Some indicated feeling shame about being tested for COVID. Such a feeling hindered participants and their peers from signing up for testing (e.g., “One time one of my friends thought that they had it and they were like, ‘Oh, I don’t know if I’m going to tested like that’s so embarrassing.”)

#### Concerns about acquiring COVID-19 and transmitting it to close contacts

Additionally, some participants expressed concerns about becoming infected or transmitting COVID-19 to others by attending in-person classes during the pandemic (Fig 1). Fears of COVID-19 infection exacerbating pre-existing medical conditions were common among immunocompromised young adults and those with existing comorbidities (e.g., “Being immunocompromised, it’s very important to me that others, you know, adhere to the guidelines when possible because this (COVID-19) is not a disease that I would like to contract or even take home to my parents.”).

## Discussion

The present study investigated university students’ views and experiences of returning to class during the COVID-19 pandemic and the perceived impact on their mental and general health. Although most respondents reported good general health, approximately half exhibited depression, anxiety, and stress symptoms. We identified several high-risk populations for mental health symptoms, including young female respondents, respondents who identified as non-binary gender, and those with pre-existing medical conditions. Additionally, findings from our focus groups suggest that sedentary behaviors, challenges of online learning, feelings of isolation, and concerns about acquiring COVID-19 infection contributed to poor mental and general well-being among university student respondents. Broad acceptance of institutional COVID-19-related policies will require clearer interactive communication, greater transparency, and information sharing. In addition to providing a comprehensive profile of mental and general health status among university students returning to class during the pandemic, our study offers concrete suggestions for improving COVID-19-related policy at the institutional level.

The prevalence of mental health symptoms in our study is slightly higher than those reported in previous studies of students’ mental health during COVID-19, which ranged from 8.7% to 25.9% for depression, 4.2% to 25% for anxiety, and 10.5% to 25% for stress. [26–29]. Differences in study design, geographical locations, and survey questionnaires may explain some differences. However, our findings may also be due to a higher percentage of respondents who were older than 18 years (98.6%), female (58.5%), and respondents with pre-existing medical conditions (19.6%) in our cohort, since the prevalence of mental health problems increase by age and are more common among females and young adults with chronic disease [30–32]. Compared with prior academic terms, increased depression and anxiety were observed among university students during the fall/autumn 2020 term, according to a U.S. longitudinal study [33]. In a Spanish study using DASS-21, researchers reported rates of moderate-to-severe symptoms of depression, anxiety, and stress among university students during the academic year 2018–2019 were approximately 11%, 16%, and 23%, respectively [34]. This prevalence was lower than what was observed in studies during the pandemic. Collectively, these findings reveal the psychological impacts of the pandemic on university students and call for timely prevention and management to reduce adverse academic and psychosocial outcomes, including psychiatric illness among students.

The present study identified subgroups of students who were likely to develop mental and physical health symptoms. Compared to male respondents, female respondents and respondents who identified as non-binary gender were significantly more likely to report mental health symptoms. The association between female gender and poor mental health was less surprising as this is consistent with earlier studies among students [27, 28, 35, 36]. Although the number of non-binary gender respondents included in the study was too small to draw definitive conclusions, we found a trend towards a much higher prevalence of depression, anxiety, and stress symptoms among this subgroup. The higher risk of mental health symptoms among these university respondents who returned to class during the pandemic may be attributable to changes in their socio-economic circumstances, difficulties in accessing gender affirmation services, and returning home to unsafe environments during COVID-19 [37, 38]. Moreover, we found that respondents with pre-existing medical conditions were susceptible to poorer mental health and had more difficulties with daily living activities during the pandemic. This is worth the attention as the proportion of students with chronic conditions in our study was comparable to those reported in the general university student population in the U.S. [39, 40], and chronic diseases significantly impact young adults’ well-being regardless of the presence of the pandemic [41, 42]. Yet, the research literature has left largely unexplored the combined effects of chronic conditions and returning to class during COVID-19 on mental and general health among university students. Therefore, the present study fills the gap by revealing specific mental and general health problems perceived by these understudied populations during the COVID-19 pandemic.

Another prominent finding from our focus group discussions was the substantial impact of COVID-19 policy on respondents’ lifestyles and mental health, which is consistent with prior studies [43]. Social distancing, isolation, and sedentary lifestyle associated with COVID-19 policies can contribute to poor mental health in young adults [3, 43, 44]. College students in places with closed campuses and all courses switched to online reported higher levels of unhappiness and worry than those in areas with open campuses in session [43]. Students may experience stress about canceling exams and anticipated events, anxiety about the job market or an academic/financial burden, and fear of infection [3]. Loss of in-person social interaction and absence of pre-COVID-19 university routine can result in boredom and lack of innovative ideas for engaging in academic and extracurricular activities [3, 45]. In the present study, nearly 80% of respondents returned to in-person classes during Fall of 2020. Our results imply that providing in-person learning experiences at an accelerating pace without addressing underlying causes of mental health problems may not necessarily have a positive influence on students’ mental well-being.

Our study also highlights challenges in implementing specific institutional-level COVID-19 policies. Perceived higher normative pressure from family and friends was associated with increased odds of intentional non-adherence to social distancing rules during the COVID-19 pandemic [46]. Research conducted in the general population has also shown that willingness to comply with governmental COVID-19 policy was markedly lower among those with low trust in the system and are concerned about infection [47]. Further, concern about transparency in information sharing, communication of evidence, and insights into decision-making processes can affect individuals’ perceptions of an institution’s pandemic response [48]. Although the circumstances and culture of each institution are unique and academic institutions’ responses to re-opening during the pandemic may differ to accommodate particular circumstances, evidence from this study shows that clear communication about COVID-19 policy, transparency, and rapid data sharing will help enhance compliance with procedures.

### Study limitations

The following study limitations need to be acknowledged. First, we surveyed students only once, preventing us from determining causative factors for increased mental or general health symptoms. Second, most respondents were recruited from social media platforms due to high concentrations of student involvement in social groups where the advertisement was posted. This limits part of the study’s subject selection to students who were active on social media during the recruitment period. Third, although respondents were from 195 majors, four different races, and five age ranges, the study was conducted in a U.S. public university with primarily white students; thus, the sample’s representativeness might be limited. Future research may be needed to recruit a more socioeconomically and ethnically diverse sample to understand better how university students think about the impact on their health of returning to class during COVID-19. Lastly, the study quantitative phase employed a cross-sectional study design, and our findings may only reflect respondents’ health status during the pre-vaccination era of the COVID-19 pandemic. COVID-19 restriction guidance has been eased, and many students may have received the COVID-19 vaccine; thus, follow-up studies are needed to determine mental and general health changes among students throughout the pandemic.

## Conclusions

Our findings demonstrate that university students were experiencing high levels of depression, anxiety, and stress while returning to class during the COVID-19 pandemic, especially female students, gender non-binary students, and those with pre-existing medical conditions. Institutional COVID-19-related policies, including social distancing, isolation, and the switch to online learning, were closely associated with students’ mental and physical health. Broad acceptance of these policies will require clear communication with students, developing students’ trust, and acknowledging students’ experiences. As universities worldwide resume in-person learning, early intervention is necessary to address students’ mental and physical health needs. The information gained from this study will help inform student-oriented wellness programs and mental health services to mitigate potential long-term negative impacts on student education and well-being.

## Supporting information

S1 Fig Mixed methods sequential explanatory design procedures. (DOCX)

S2 Fig Flow diagram of survey and focus group study recruitment. (DOCX)

S1 Method Overview of the social media recruitment process. (DOCX)

S2 Method Focus group discussion guide. (DOCX)

S3 Method NVivo qualitative analysis procedure. (DOCX)

S1 Table Example of interpretive description coding and analysis. (DOCX)

S2 Table Demographics of focus group population by gender. (DOCX)

S3 Table Prevalence of symptoms of DASS-21 and EQ-5D scales stratified by focus group respondents’ characteristics. (DOCX)

S4 Table Median and interquartile ranges of EQ-5D VAS scores by survey respondents’ characteristics. (DOCX)

S5 Table Adjusted multivariable regression analysis of DASS-21 associated with characteristics of survey respondents. (DOCX)

S6 Table Unadjusted analysis of DASS-21 associated with characteristics of survey respondents. (DOCX)

S7 Table Unadjusted analysis of EQ-5D scales associated with characteristics of survey respondents. (DOCX)

S8 Table Adjusted multivariable regression analysis of EQ-5D scales associated with characteristics of survey respondents. (DOCX)

S9 Table Multiple linear regression analysis of factors associated with overall health score (EQ-5D-VAS) in survey respondents. (DOCX)

S10 Table Sensitivity analysis of factors associated with mental health and overall health score, restricted to survey respondents attending in-person class only. (DOCX)

S11 Table Key themes in respondents’ feedback from focus group discussion. (DOCX)

S12 Table Checklist for Reporting Results of Internet E-Surveys (CHERRIES). (PDF)

## Abbreviations

EQ-5D-5L: EuroQol 5-Dimension 5-Level
DASS-21: Depression, Anxiety and Stress Scale 21
