# Supplementary figures and images for: A mixed-methods approach to understand university students’ perceived impact of returning to class during COVID-19 on their mental and general health
Source: PLoS One. 2023 Jan 3;18(1):e0279813. doi: 10.1371/journal.pone.0279813 (PMC9810175; doi:10.1371/journal.pone.0279813)

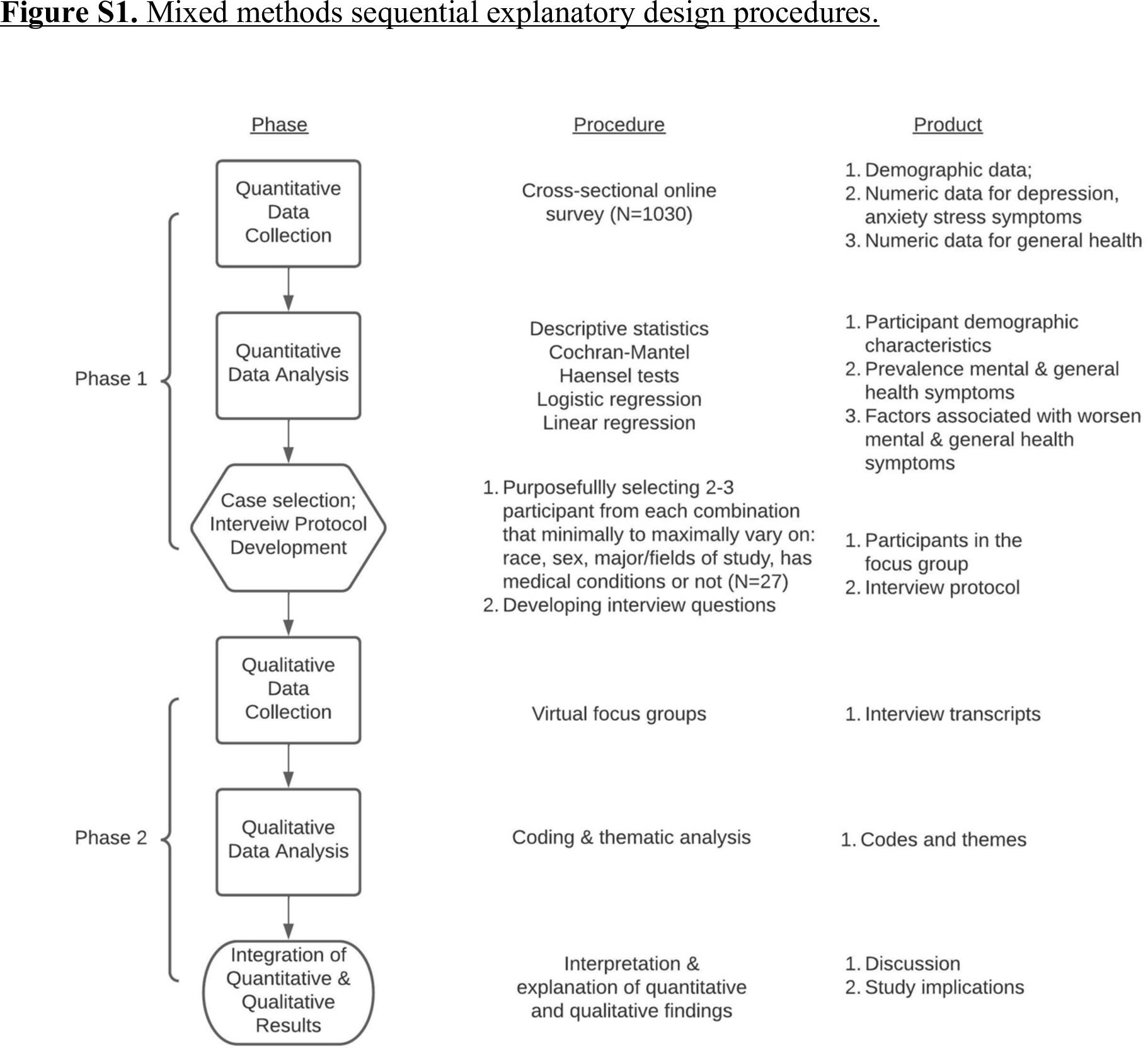

Supplement: S1 Fig — (DOCX) [file pone.0279813.s001.docx]

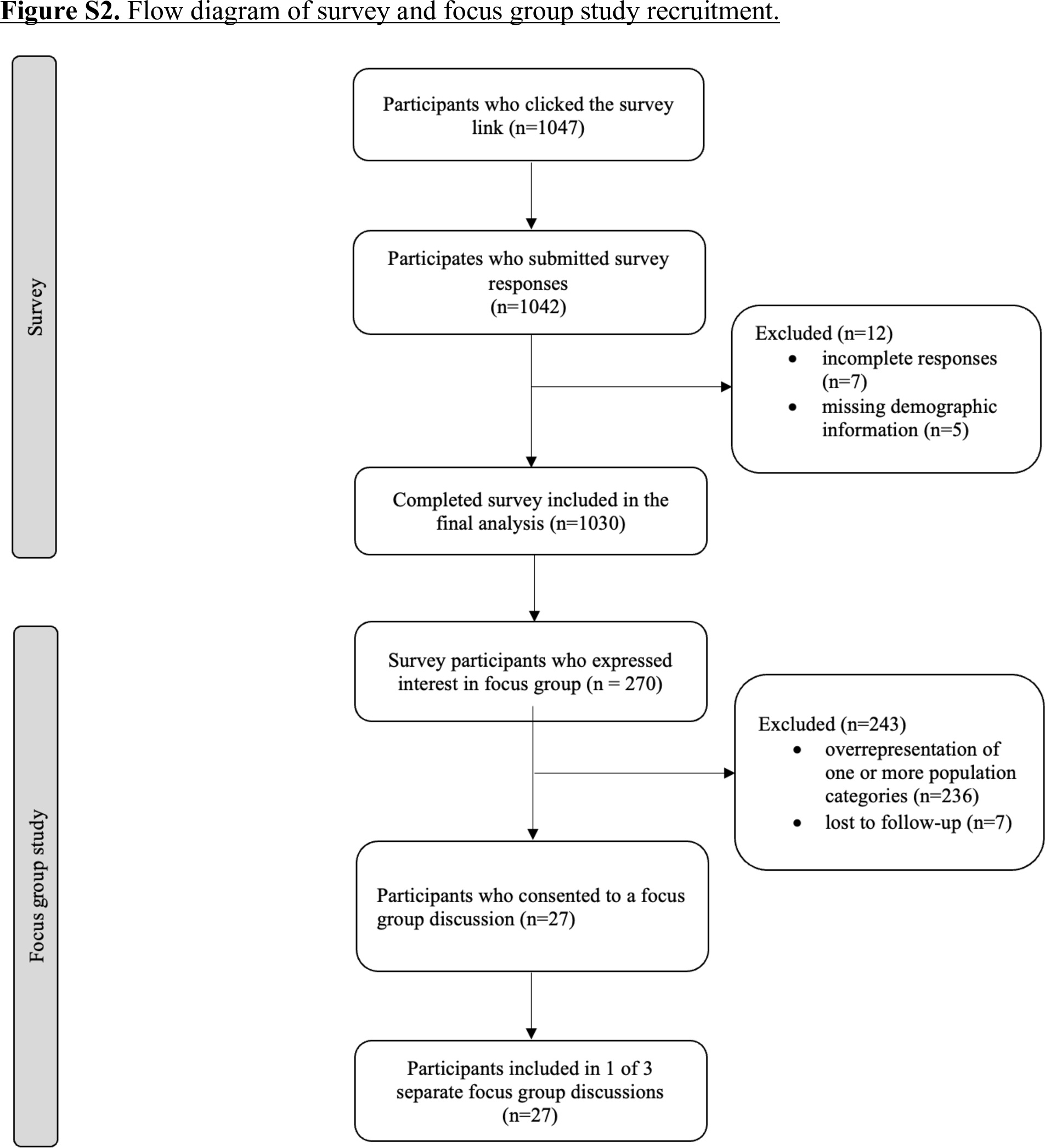

Supplement: S2 Fig — (DOCX) [file pone.0279813.s002.docx]
