# Supplementary material for: A mixed-methods approach to understand university students’ perceived impact of returning to class during COVID-19 on their mental and general health
Source: PLoS One. 2023 Jan 3;18(1):e0279813. doi: 10.1371/journal.pone.0279813 (PMC9810175; doi:10.1371/journal.pone.0279813)
Supplement: S1 Method — (DOCX) [file pone.0279813.s003.docx]

**Method S1**: Overview of the social media recruitment process

We conducted an informal social media advertising campaign to promote our study and attract more participants with diverse backgrounds. The targeted population was young adults who had attended a public university in the Midwest, U.S., during Fall 2020. A general advertisement template was created for use. Edits were made according to the tools the specific social media platform provided to help interact with its users. The formatting for the advertisement included: 1) headline; 2) original graphic; 3) description; 4) website URL to the Qualtrics survey; and 5) invitation to participate. We considered a range of social media platforms to advertise on, prioritizing those with higher concentrations of targeted audience and higher usage frequencies by our targeted audience. Final platforms chosen included Reddit, Instagram, and Facebook. Advertisements were also posted on several micro-social networks and group-chatting platforms that specifically targeted students enrolled in the university, including GroupMe, Discord, and Piazza. In addition to the use of social media platforms, we incorporated electronic mailing lists as a part of our campaign. The mailing list of 5000 students was randomly generated by the university registrar’s office, focusing on both undergraduate and graduate students of all races/ethnicities, university-wide student groups and extracurricular organizations. All materials and procedures, including the Qualtrics survey and advertisement content, underwent IRB approval.

The most interactive social media campaign in this study occurred through Reddit and Facebook groups (e.g., university “Class of..." groups). We also observed a high willingness of participations on academically oriented platforms that were popular among students enrolled in the university, such as GroupMe and Piazza. Even though these micro-social networks proved effective, concern for bias and skewing in the data arose a week into the campaign, as only students enrolled in certain academic programs, classes, or study groups were allowed to be enrolled on these platforms. This concern was evidenced by a higher proportion of participants self-identified as Asian and from the College of Engineering. To address this in a timely fashion, we added Instagram as an additional social media platform to promote our study to a wider range of students and multidisciplinary organizations.

Trained research assistants monitored our study advertisements on the above social media platforms for the duration of the campaign for any commentary. Comments posted under the advertised posts or hashtag were screened by the research team and were removed if determined to be based on bias, personal dislike, or personal animosity. The social media recruitment process ended on November 20, 2020, and the Qualtrics survey was closed on December 1, 2020, per study protocol.
