# Supplementary material for: A mixed-methods approach to understand university students’ perceived impact of returning to class during COVID-19 on their mental and general health
Source: PLoS One. 2023 Jan 3;18(1):e0279813. doi: 10.1371/journal.pone.0279813 (PMC9810175; doi:10.1371/journal.pone.0279813)
Supplement: S2 Method — (DOCX) [file pone.0279813.s004.docx]

**Method S2:** Focus group discussion guide.

Hello, we are the research team members from the study titled "A Mixed-Methods Approach to Understand University Students’ Perceived Impact of Returning to School During COVID-19 on Their Mental Health and General Well-Being ", we wanted to welcome you to participate in our focus group discussion.

When you filled out our survey a few weeks ago, you were given an information sheet describing our study. You indicated agreement to participate in the focus group discussion part of the study and provided us an email contact. This is the virtual focus group study, and we will ask you four questions about your perceptions and experiences attending classes this fall. Our focus group will last about 1-hour and you will not be asked to give your names, class or major or any other self-identification. Additionally, you are not required to turn on your camara throughout the focus group discussion. Although we, the researchers, will safeguard the confidentiality of the discussion to the best of our ability, the nature of focus groups prevents us from guaranteeing that other members of the group will do so. Please respect the confidentiality of the other members of the group by not repeating what is said in the focus group to others and be aware that other members of the group may not respect your confidentiality. The focus group will be audio-recorded to accurately capture what is said. If you chose to participate in the focus group, you may request the recording be paused at any time. You may choose how much or how little you want to speak during the group. You may also choose to leave the focus group at any time.

Just to confirm before we start, do you still agree to participate in this 1-hour virtual focus group discussion by answering 4 questions? Only if you confirm your willingness to participate in the focus group, will you be allowed to remain in the focus group and continue in our next section.

Thank you for confirming your willingness to proceed. Next, we will be asking you a few questions. You may choose not to answer the questions if you are not willing to. You will be compensated with a $10 Amazon E-gift card for your participation in this focus group discussion.

1. What are the psychological impacts of social distancing and attending school in-person or remotely as experienced by you this Fall? How does attending school during the COVID-19 pandemic affect your overall health?

1. What are your views on the university's COVID-19 measures and its communication around COVID-19-related policy?
2. Do you feel that the policy provided protection for the students and faculty?

1. What are your current experiences of adherence in relation to the university's COVID-19 measures this Fall?

1. What are your suggestions about future policy and strategies to help protect your general health and mental well-being while attending school in Spring 2021?
