# Supplementary material for: A mixed-methods approach to understand university students’ perceived impact of returning to class during COVID-19 on their mental and general health
Source: PLoS One. 2023 Jan 3;18(1):e0279813. doi: 10.1371/journal.pone.0279813 (PMC9810175; doi:10.1371/journal.pone.0279813)
Supplement: S3 Method — (DOCX) [file pone.0279813.s005.docx]

**Method S3:** NVivo Qualitative Analysis Procedure.

The three focus group discussions were transcribed by three trained research assistants independently using the verbatim method, and the transcriptions were reviewed by the study principal investigator for content accuracy. Verified transcriptions were then uploaded to NVivo, version 12.3, a qualitative data analysis software used to extract, code, store and identify content themes. Two researchers (QD and EW) analyzed the transcripts, developed the codebook, and identified the themes through content and thematic framework analysis. Thematic framework analysis was used to sift, chart and sort data in accordance with key issues and themes following five steps: familiarization, identification of a thematic framework, creation of indexing, creation of charting and mapping, and interpretation (1).

Step 1. Before coding independently, both researchers began by reading the transcripts closely to become familiar with the qualitative data. They also made notes of any statements which later could be used to construct preliminary codes.

Step 2. the two researchers coded the transcripts independently and generated initial list of theme codes. They then met to discuss codes and compared for consistency. A code may be found in more than one post. An initial set of codes to use were developed. In sequential research meetings, researchers discussed any overarching themes across coded data and resolved any discrepancies between themes. The code book was updated accordingly. All preliminary codes were considered within the context of the research questions which were to identify the underlying causes and factors influencing students’ mental and general health and to examine how these factors could explain the high prevalence of symptoms of depression, anxiety and stress among university students returning to school during the COVID-19 pandemic.

Step 3. Each post was analyzed and reviewed in an iterative process in isolation from the previous quote to ensure the consistent application of codes and themes. This iterative process was repeated three times until the entire transcripts were coded and both researchers acknowledged the thematic analysis of transcripts provided sufficient information to generate meaningful insights into the University students’ community of who attended class during COVID. The two researchers developed names and definitions for each theme code. They then identified similarities and discrepancies across codes to develop the coding framework and organize codes into theme content categories and subcategories. The coding framework was used to divide quotes into a hierarchal system of themes and subthemes.

Step 4. The two researchers rearranged the quotes and examined the quotes and themes systematically. A spreadsheet was used to generate a matrix and to lay out codes, quotes, themes/subthemes, and summarized study findings.

Step 5. The matrix, thematic map and all themes were shared and discussed among the multi-disciplinary teams to ensure consistency within the team with interpretation. Themes were further refined, and interpretation of findings were discussed before writing the results.
