# Supplementary material for: A mixed-methods approach to understand university students’ perceived impact of returning to class during COVID-19 on their mental and general health
Source: PLoS One. 2023 Jan 3;18(1):e0279813. doi: 10.1371/journal.pone.0279813 (PMC9810175; doi:10.1371/journal.pone.0279813)
Supplement: S1 Table — (DOCX) [file pone.0279813.s006.docx]

**Table S1**. Example of interpretive description coding and analysis.

| **Transcript Excerpt** | **Early codes applied** |
| --- | --- |
| 13: I don’t know, I think the thing I’ve experienced a lot as well is a lot of, it’s hard to communicate online. A lot my professors have been somewhat, not intentionally, but vague in assignments or it’s hard to get a hold of people. It’s really easy to say that you’re going to have a Zoom meeting, and I have a few TA’s that simply just don’t get on the Zoom meeting for office hours, so just, I feel like it’s a lot easier in person when you’re like face to face with someone to walk up and ask a question versus with a Zoom meeting it’s hard to stay behind because a lot of professors just end the zoom meeting. So, a lot of confusion in terms of assignments and what’s going on, and that alone is leading to a lot of stress, but then also along with that I feel like a lot of professors are not really clear in standing of classes. I personally, I have no problem sharing this, I’m an engineer, my classes are hard, and the averages are particularly low this semester on a lot of exams and stuff, and I feel like professors aren’t really addressing that and they’re also not suggesting what we can do to improve or offering more office hours despite the lack of them anywhere. So just the whole situation of being online and not in person, it’s a lot harder to get the help that you need, and then not being able to get help increases my stress, and then not doing well just kind of decreases my motivation. | Communication difficulties  Barriers in online learning:   - Lack of contact with professors - Getting help on work (office hours or suggestions to improvement on work) - Unclarity with assignments   Stress-inducing learning environment  Decreases to motivation  Lack of support from teachers and in classwork |
| 11: …So much of the university’s emphasis is on individual behavior, and that’s important, it’s very important to wear your mask, but I think that what is really missing from the university’s whole approach is this sense of what is our responses as an institution, what are the graduated steps that we take when this or this or this happens. So right now, it’s basically well, we’ll all wear our masks and come back to campus, and that’s just how it’s going to be. But that really doesn’t take into account different levels of spread, and levels of dangers, so is there is a phase at which, when we hit a certain metric, that all instruction goes online for X amount of time or is there a phase at which certain kinds of labs have to go remote. Making that plan public, and those gradations really clear would be a major, major right step in the university’s pandemic response, and I think, in making, giving students, faculty, and staff confidence in Purdue’s ability to care for people on its campus. I mean, it's hard to make those decision because that has financial implications, those financial implications might not be pretty, but on the other hand it’s the right thing to do. | Individual vs institutional responsibility  Increased institutional accountability  Consider levels of spread and other measures  Impacts of more transparent pandemic response:   - Greater confidence instilled in students/faculty - May have financial consequences |
| **Iterative reflections *(themes italicized)*** | |
| While returning to school during the COVID-19 pandemic, students mainly lived sedentary lifestyles. They also experienced disruptions to sleep, eating patterns, exercise, and general lifestyle (attending school during COVID-19 is associated with unhealthy behaviors and poor health). Students also mentioned other difficulties while attending school during COVID-19 mostly related to their new learning environment. Some students experienced challenges in getting help from or communicating with professors, having fewer interactions with colleagues, and spending more time doing schoolwork alone. All these lead to more  feelings of isolation (perceived challenges of online learning and increased feelings of isolation).  Students who attended classes on campus felt that their peers were taking steps to prevent COVID-19 transmission, such as mask-wearing or sanitizing. But they expressed frustration that there wasn’t enough institutional accountability in taking preventative COVID-19 measures, such as accounting for levels of spread in different spaces (demands for COVID-19 policy reform and greater transparency of university COVID-19 statistics). On the other hand, some students who had in-person classes felt that their peers were not adhering enough to COVID-19 testing regulations, social distancing, or abiding by mask-wearing rules (difficulties in adhering to COVID-19 related policies and protocols implemented by the university). Because of this, immunocompromised students expressed concerns about being exposed to COVID-19 or spreading it to peers or family members (concerns about acquiring COVID-19 and transmitting it to close contacts). | |
