# Supplementary material for: A mixed-methods approach to understand university students’ perceived impact of returning to class during COVID-19 on their mental and general health
Source: PLoS One. 2023 Jan 3;18(1):e0279813. doi: 10.1371/journal.pone.0279813 (PMC9810175; doi:10.1371/journal.pone.0279813)
Supplement: S2 Table — (DOCX) [file pone.0279813.s007.docx]

**Table S2.** Demographics of focus group population by gender.

| Characteristic | | **Total**  **(n=27)** |
| --- | --- | --- |
| **Age range, n (%)** | |  |
|  | 15-17 | 1 (4) |
|  | 18-24 | 18 (67) |
|  | 25-29 | 6 (22) |
|  | >30 | 2 (7) |
| **Race, n (%)** | |  |
|  | White | 13 (48) |
|  | Asian | 8 (30) |
|  | Black | 1 (4) |
|  | More than one race or other | 5 (19) |
| **Level of education, n (%)** | |  |
|  | Undergraduate | 15 (56) |
|  | Graduate | 12 (44) |
| **Has in-person classes for Fall 2020? n (%)** | |  |
|  | Yes | 19 (70) |
|  | No | 8 (30) |
| **Living arrangement, n (%)** | |  |
|  | Off-campus housing | 19 (70) |
|  | University residences | 7 (26) |
|  | Staying at home | 1 (4) |
|  | Currently outside of the United States | 0 (0) |
| **Has medical conditions? n (%)** | |  |
|  | No | 20 (74) |
|  | Yes | 7 (26) |
| **Work, n (%)** | |  |
|  | Part-time | 17 (63) |
|  | Full-time | 2 (7) |
|  | Unemployed | 8 (30) |
| **How did you hear about our study? n (%)** | |  |
|  | Electronic mailing list | 19 (70) |
|  | Social networking platform | 6 (22) |
|  | Word of mouth (ex. peers, friends) | 2 (7) |
