# Supplementary material for: A mixed-methods approach to understand university students’ perceived impact of returning to class during COVID-19 on their mental and general health
Source: PLoS One. 2023 Jan 3;18(1):e0279813. doi: 10.1371/journal.pone.0279813 (PMC9810175; doi:10.1371/journal.pone.0279813)
Supplement: S3 Table — (DOCX) [file pone.0279813.s008.docx]

**Table S3.** Prevalence of symptoms of DASS-21 and EQ-5D scales, stratified by focus group respondents’ characteristics.

**S3.1**. DASS-21

| Variables | | Depression | | | Anxiety |  |  | Stress | | |
| --- | --- | --- | --- | --- | --- | --- | --- | --- | --- | --- |
|  |  | Normal, n (%) | Mild, n (%) | Moderate to extremely severe, n (%) | Normal, n (%) | Mild, n (%) | Moderate to extremely severe, n (%) | Normal, n (%) | Mild, n (%) | Moderate to extremely severe, n (%) |
| Overall | | 6 (24) | 3 (12) | 16 (64) | 9 (33) | 4 (15) | 14 (52) | 9 (35) | 5 (19) | 12 (46) |
| Gender | |  |  |  |  |  |  |  |  |  |
|  | Male | 0 (0) | 4 (80) | 2 (33) | 1 (17) | 3 (50) | 3 (23) | 4 (31) | 6 (46) | 6 (46) |
|  | Female | 3 (15) | 12 (60) | 7 (33) | 3 (14) | 11 (52) | 6 (46) | 1 (8) | 6 (46) | 6 (46) |
| Race | |  |  |  |  |  |  |  |  |  |
|  | White | 0 (0) | 9 (69) | 4 (31) | 2 (15) | 7 (54) | 3 (19) | 5 (31) | 8 (50) | 8 (50) |
|  | Non-white | 3 (25) | 7 (58) | 5 (36) | 2 (14) | 7 (50) | 6 (46) | 0 (0) | 7 (54) | 7 (54) |
| Age range | |  |  |  |  |  |  |  |  |  |
|  | 15-24 | 3 (17) | 12 (67) | 6 (32) | 3 (16) | 10 (53) | 6 (33) | 3 (17) | 9 (50) | 9 (50) |
|  | ≥25 | 0 (0) | 4 (57) | 3 (38) | 1 (13) | 4 (50) | 3 (38) | 2 (25) | 3 (38) | 3 (38) |
| Education level | |  |  |  |  |  |  |  |  |  |
|  | Undergraduate | 1 (8) | 9 (69) | 5 (33) | 1 (7) | 9 (60) | 6 (40) | 2 (13) | 7 (47) | 7 (47) |
|  | Graduate | 2 (17) | 7 (58) | 4 (33) | 3 (25) | 5 (42) | 3 (27) | 3 (27) | 5 (45) | 5 (45) |
| Living arrangement | |  |  |  |  |  |  |  |  |  |
|  | Living in UR^[[1]](#footnote-1)^ | 1 (14) | 5 (71) | 4 (57) | 1 (14) | 2 (29) | 4 (57) | 1 (14) | 2 (29) | 2 (29) |
|  | Other | 2 (11) | 11 (61) | 5 (25) | 3 (15) | 12 (60) | 5 (26) | 4 (21) | 10 (53) | 10 (53) |
| Work status | |  |  |  |  |  |  |  |  |  |
|  | Employed | 1 (6) | 11 (65) | 5 (26) | 2 (11) | 12 (63) | 5 (28) | 4 (22) | 9 (50) | 9 (50) |
|  | Non-employed | 2 (25) | 5 (63) | 4 (50) | 2 (25) | 2 (25) | 4 (50) | 1 (13) | 3 (38) | 3 (38) |
| Has in-person class(es) for Fall 2020? (Y/N)^[[2]](#footnote-2)^ | |  |  |  |  |  |  |  |  |  |
|  | Yes | 3 (18) | 11 (65) | 6 (32) | 3 (16) | 10 (53) | 6 (33) | 3 (17) | 9 (50) | 9 (50) |
|  | No | 0 () | 5 (63) | 3 (38) | 1 (13) | 4 (50) | 3 (38) | 2 (25) | 3 (38) | 3 (38) |
| Has medical conditions? (Y/N) | |  |  |  |  |  |  |  |  |  |
|  | Yes | 0 (0) | 5 (71) | 2 (29) | 0 (0) | 5 (71) | 1 (14) | 3 (43) | 3 (43) | 3 (43) |
|  | No | 3 (17) | 11 (61) | 7 (35) | 4 (20) | 9 (45) | 8 (42) | 2 (11) | 9 (47) | 9 (47) |

**S3.2** EQ-5D.

| Characteristic | | Mobility | Self-care | Usual activities | | | Pain/discomfort | | |
| --- | --- | --- | --- | --- | --- | --- | --- | --- | --- |
|  |  | Normal, n (%) | Normal, n (%) | Normal, n (%) | Mild, n (%) | Moderate to extremely severe, n (%) | Normal, n (%) | Mild, n (%) | Moderate to extremely severe, n (%) |
| Overall | | 27 (100.0) | 27 (100.0) | 17 (63) | 3 (11) | 7 (26) | 18 (67) | 8 (30) | 1 (3) |
| Gender | |  |  |  |  |  |  |  |  |
|  | Male | 6 (100.0) | 6 (100.0) | 5 (83) | 0 (0) | 1 (17) | 5 (83) | 1 (17) | 0 (0) |
|  | Female | 21 (100.0) | 21 (100.0) | 12 (57) | 3 (14) | 6 (29) | 13 (62) | 7 (33) | 1 (5) |
| Race | |  |  |  |  |  |  |  |  |
|  | White | 13 (100.0) | 13 (100.0) | 7 (54) | 3 (23) | 3 (23) | 7 (54) | 6 (46) | 0 (0) |
|  | Non-white | 14 (100.0) | 14 (100.0) | 10 (71) | 0 (0) | 4 (29) | 11 (79) | 2 (14) | 1 (7) |
| Age range | |  |  |  |  |  |  |  |  |
|  | 15-24 | 19 (100.0) | 19 (100.0) | 11 (58) | 3 (16) | 5 (26) | 12 (63) | 6 (32) | 1 (5) |
|  | ≥25 | 8 (100.0) | 8 (100.0) | 6 (75) | 0 (0) | 2 (25) | 6 (75) | 2 (25) | 0 (0) |
| Education level | |  |  |  |  |  |  |  |  |
|  | Undergraduate | 15 (100.0) | 15 (100.0) | 9 (60) | 1 (7) | 5 (33) | 11 (73) | 3 (20) | 1 (7) |
|  | Graduate | 12 (100.0) | 12 (100.0) | 8 (67) | 2 (17) | 2 (17) | 7 (58) | 5 (42) | 0 (0) |
| Living arrangement | |  |  |  |  |  |  |  |  |
|  | Living in UR^[[3]](#footnote-3)^ | 7 (100.0) | 7 (100.0) | 5 (71) | 0 (0) | 2 (29) | 4 (57) | 2 (29) | 1 (14) |
|  | Other | 20 (100.0) | 20 (100.0) | 12 (60) | 3 (15) | 5 (25) | 14 (70) | 6 (30) | 0 () |
| Work status | |  |  |  |  |  |  |  |  |
|  | Employed | 19 (100.0) | 19 (100.0) | 11 (58) | 3 (16) | 5 (26) | 13 (68) | 6 (32) | 0 (0) |
|  | Non-employed | 8 (100.0) | 8 (100.0) | 6 (75) | 0 (0) | 2 (25) | 5 (63) | 2 (25) | 1 (13) |
| Has in-person class(es) for Fall 2020? (Y/N)^[[4]](#footnote-4)^ | |  |  |  |  |  |  |  |  |
|  | Yes | 19 (100.0) | 19 (100.0) | 13 (68) | 3 (16) | 3 (16) | 12 (63) | 6 (32) | 1 (5) |
|  | No | 8 (100.0) | 8 (100.0) | 4 (50) | 0 (0) | 4 (50) | 6 (75) | 2 (25) | 0 (0) |
| Has medical conditions? (Y/N) | |  |  |  |  |  |  |  |  |
|  | Yes | 7 (100.0) | 7 (100.0) | 4 (57) | 1 (14) | 2 (29) | 4 (57) | 3 (43) | 0 (0) |
|  | No | 20 (100.0) | 20 (100.0) | 13 (65) | 2 (10) | 5 (25) | 14 (70) | 5 (25) | 1 (5) |

**S3.2.** continued.

| Characteristic | | Anxiety/depression | | |
| --- | --- | --- | --- | --- |
|  |  | Normal, n (%) | Mild, n (%) | Moderate to extremely severe, n (%) |
| Overall | | 2 (7) | 6 (22) | 19 (71) |
| Gender | |  |  |  |
|  | Male | 1 (17) | 1 (17) | 4 (66) |
|  | Female | 1 (5) | 5 (24) | 15 (71) |
| Race | |  |  |  |
|  | White | 0 (0) | 3 (23) | 10 (77) |
|  | Non-white | 2 (14) | 3 (21) | 9 (64) |
| Age range | |  |  |  |
|  | 15-24 | 2 (11) | 4 (21) | 13 (68) |
|  | ≥25 | 0 (0) | 2 (25) | 6 (75) |
| Education level | |  |  |  |
|  | Undergraduate | 2 (13) | 3 (20) | 10 (67) |
|  | Graduate | 0 (0) | 3 (25) | 9 (75) |
|  | Other | 1 (5) | 6 (30) | 13 (65) |
| Living arrangement | |  |  |  |
|  | Living in UR^[[5]](#footnote-5)^ | 2 (29) | 2 (29) | 3 (43) |
|  | Other | 0 (0) | 4 (20) | 16 (80) |
| Work status | |  |  |  |
|  | Employed | 1 (5) | 3 (16) | 15 (79) |
|  | Non-employed | 1 (13) | 3 (38) | 4 (50) |
| Has in-person class(es) for Fall 2020? (Y/N)^[[6]](#footnote-6)^ | |  |  |  |
|  | Yes | 2 (11) | 4 (21) | 13 (68) |
|  | No | 0 (0) | 2 (25) | 6 (75) |
| Has medical conditions? (Y/N) | |  |  |  |
|  | Yes | 0 (0) | 1 (14) | 6 (86) |
|  | No | 2 (10) | 5 (25) | 13 (65) |

1. UR: university residences [↑](#footnote-ref-1)
2. Y/N: yes/no [↑](#footnote-ref-2)
3. UR: university residences [↑](#footnote-ref-3)
4. Y/N: yes/no [↑](#footnote-ref-4)
5. UR: university residences [↑](#footnote-ref-5)
6. Y/N: yes/no [↑](#footnote-ref-6)
