# Supplementary material for: A mixed-methods approach to understand university students’ perceived impact of returning to class during COVID-19 on their mental and general health
Source: PLoS One. 2023 Jan 3;18(1):e0279813. doi: 10.1371/journal.pone.0279813 (PMC9810175; doi:10.1371/journal.pone.0279813)
Supplement: S4 Table — (DOCX) [file pone.0279813.s009.docx]

**Table S4.** Median and interquartile ranges of EQ-5D VAS scores by survey respondents’ characteristics.

| Characteristic | Median (IQR)^[[1]](#footnote-1)^ |
| --- | --- |
| Overall | 75.0 (60.0-85.0) |
| Gender |  |
| Male | 75.0 (61.0-85.0) |
| Female | 75.0 (60.0-85.0) |
| Other | 74.0 (59.0-82.5) |
| Race |  |
| White | 75.0 (60.0-85.0) |
| Non-white | 79.5 (65.0-88.3) |
| Age range |  |
| 15-24 | 75.0 (61.3-90.0) |
| ≥ 25 | 75.0 (60.0-85.0) |
| Education level |  |
| Undergraduate | 75.0 (60.0-85.0) |
| Graduate | 75.0 (61.0-88.0) |
| Living arrangement |  |
| Living in UR^[[2]](#footnote-2)^ | 75.0 (85.0-61.0) |
| Not living in  UR | 75.0 (60.0-85.3) |
| Work status |  |
| Employed | 75.0 (80.0-85.0) |
| Non-employed | 75.0 (61.0-85.0) |
| Has in-person classes for Fall 2020? (Y/N)^[[3]](#footnote-3)^ |  |
| Yes | 75.0 (60.0-85.0) |
| No | 75.0 (60.0-85.0) |
| Has medical conditions? (Y/N) |  |
| Yes | 68.5 (50.0-80.0) |
| No | 77.0 (65.0-86.0) |

1. The decision to use the median and interquartile range of the VAS scores to measure the data’s central tendency was determined based on the distribution of the scores. [↑](#footnote-ref-1)
2. UR: university residences [↑](#footnote-ref-2)
3. Y/N: yes/no [↑](#footnote-ref-3)
