# Supplementary material for: A mixed-methods approach to understand university students’ perceived impact of returning to class during COVID-19 on their mental and general health
Source: PLoS One. 2023 Jan 3;18(1):e0279813. doi: 10.1371/journal.pone.0279813 (PMC9810175; doi:10.1371/journal.pone.0279813)
Supplement: S5 Table — (DOCX) [file pone.0279813.s010.docx]

**Table S5.** Adjusted multivariable regression analysis of DASS-21 associated with characteristics of survey respondents.

| Characteristic | | Depression | | Anxiety | | Stress | |
| --- | --- | --- | --- | --- | --- | --- | --- |
|  |  | AOR (95% CI) | *P* value | AOR (95% CI) | *P* value | AOR (95% CI) | *P* value |
| Gender | |  |  |  |  |  |  |
|  | Female | 1.56 (1.18-2.05) | <.001* | 1.82 (1.37-2.42) | <.001* | 1.80 (1.36-2.38) | <.001* |
|  | Other | 11.53 (1.46-90.96) | .002* | 5.13 (1.51-17.38) | .005* | 3.93 (1.26-13.62) | .018* |
|  | Male | 1 [Reference] |  | 1 [Reference] |  | 1 [Reference] |  |
| Race | |  |  |  |  |  |  |
|  | Non-white | 0.74 (0.55-0.99) | .043* | 0.71 (0.52-0.95) | .021* | 0.58 (0.42-0.78) | <.001* |
|  | White | 1 [Reference] |  | 1 [Reference] |  | 1 [Reference] |  |
| Age range | |  |  |  |  |  |  |
|  | ≥ 25 | 0.89 (0.47-1.68) | .716 | 1.14 (0.60-2.17) | .690 | 0.64 (0.34-1.22) | .178 |
|  | 15-24 | 1 [Reference] |  | 1 [Reference] |  | 1 [Reference] |  |
| Work status | |  |  |  |  |  |  |
|  | Employed | 1.11 (0.83-1.49) | .466 | 1.24 (0.93-1.66) | .145 | 1.23 (0.91-1.65) | .172 |
|  | Unemployed | 1 [Reference] |  | 1 [Reference] |  | 1 [Reference] |  |
| Living arrangement | |  |  |  |  |  |  |
|  | Living in UR^[[1]](#footnote-1)^ | 0.78 (0.58-1.05) | .0956 | 0.79 (0.59-1.06) | .120 | 0.65 (0.48-0.88) | .005* |
|  | Not living in UR | 1 [Reference] |  | 1 [Reference] |  | 1 [Reference] |  |
| Education level | |  |  |  |  |  |  |
|  | Undergraduate | 1.23 (0.69-2.15) | .4666 | 1.60 (0.91-2.81) | .095 | 1.00 (0.57-1.75) | 1.000 |
|  | Graduate | 1 [Reference] |  | 1 [Reference] |  | 1 [Reference] |  |
| Has in-person classes for Fall 2020? (Y/N)^[[2]](#footnote-2)^ | |  |  |  |  |  |  |
|  | Yes | 1.15 (0.81-1.65) | 0.4511 | 1.16 (0.81-1.67) | 0.4205 | 0.76 (0.53-1.09) | 0.1340 |
|  | No | 1 [Reference] |  | 1 [Reference] |  | 1 [Reference] |  |
| Has medical conditions? (Y/N) | |  |  |  |  |  |  |
|  | Yes | 1.59 (1.122.26) | 0.0086 | 2.15 (1.55-3.01) | <.0001* | 2.06 (1.46-2.91) | <.0001* |
|  | No | 1 [Reference] |  | 1 [Reference] |  | 1 [Reference] |  |

1. UR: university residences [↑](#footnote-ref-1)
2. Y/N: yes/no

   * significant at ≤0.05

   * significant at ≤0.05 [↑](#footnote-ref-2)
