# Supplementary material for: A mixed-methods approach to understand university students’ perceived impact of returning to class during COVID-19 on their mental and general health
Source: PLoS One. 2023 Jan 3;18(1):e0279813. doi: 10.1371/journal.pone.0279813 (PMC9810175; doi:10.1371/journal.pone.0279813)
Supplement: S6 Table — (DOCX) [file pone.0279813.s011.docx]

**Table S6.** Unadjusted analysis of DASS-21 associated with characteristics of survey respondents.

| Characteristic | | DASS  Depression | DASS Anxiety | DASS  Stress |
| --- | --- | --- | --- | --- |
|  |  | OR (95% CI) | OR (95% CI) | OR (95% CI) |
| Gender | |  |  |  |
|  | Male | 1 [Reference] | 1 [Reference] | 1 [Reference] |
|  | Female | 1.71  (1.32-2.21) | 2.07  (1.59-2.70) | 2.07  (1.59-2.71) |
|  | Other | 11.65 (2.26-213.08) | 5.43 (1.78-20.09) | 3.71 (1.26-12.29) |
| Race | |  |  |  |
|  | White | 1 [Reference] | 1 [Reference] | 1 [Reference] |
|  | Non-white | 0.69  (0.53-0.90) | 0.62  (0.47-0.81) | 0.58  (0.43-0.76) |
| Age range | |  |  |  |
|  | ≥25 | 0.83  (0.56-1.24) | 0.91  (0.61-1.36 | 0.97  (0.64-1.45) |
|  | 15-24 | 1 [Reference] | 1 [Reference] | 1 [Reference] |
| Education level | |  |  |  |
|  | Undergraduate | 1.11  (0.79-1.56) | 1.13  (0.80-1.59) | 0.86  (0.60-1.21) |
|  | Graduate | 1 [Reference] | 1 [Reference] | 1 [Reference] |
| Living arrangement | |  |  |  |
|  | Not Living in UR^[[1]](#footnote-1)^ | 1 [Reference] | 1 [Reference] | 1 [Reference] |
|  | Living in UR | 0.85  (0.66-1.09) | 0.855  (0.67-1.09) | 0.64  (0.49-0.83) |
| Work status | |  |  |  |
|  | Employed | 1.23  (0.95-1.59) | 1.38  (1.07-1.78) | 1.49  (1.15-1.94) |
|  | Non-employed | 1 [Reference] | 1 [Reference] | 1 [Reference] |
| Has in-person class(es) for Fall 2020? (Y/N)^[[2]](#footnote-2)^ | |  |  |  |
|  | Yes | 1.26  (0.93-1.70) | 1.29  (0.95-1.76) | 0.86  (0.64-1.17) |
|  | No | 1 [Reference] | 1 [Reference] | 1 [Reference] |
| Has medical conditions? (Y/N) | |  |  |  |
|  | No | 1 [Reference] | 1 [Reference] | 1 [Reference] |
|  | Yes | 1.82 (1.31-2.57) | 2.48  (1.81-3.43) | 2.42  (1.75-3.38) |

1. UR: university residences [↑](#footnote-ref-1)
2. Y/N: yes/no [↑](#footnote-ref-2)
