# Supplementary material for: A mixed-methods approach to understand university students’ perceived impact of returning to class during COVID-19 on their mental and general health
Source: PLoS One. 2023 Jan 3;18(1):e0279813. doi: 10.1371/journal.pone.0279813 (PMC9810175; doi:10.1371/journal.pone.0279813)
Supplement: S7 Table — (DOCX) [file pone.0279813.s012.docx]

**Table S7.** Unadjusted analysis of EQ-5D scales associated with characteristics of survey respondents.

| Characteristic | | Mobility | Self-care | Usual activities | Pain/discomfort | Anxiety/depression |
| --- | --- | --- | --- | --- | --- | --- |
|  |  | OR (95% CI) | OR (95% CI) | OR (95% CI) | OR (95% CI) | OR (95% CI) |
| Gender | |  |  |  |  |  |
|  | Female | 3.81  (1.02-24.74) | 3.14  (1.16-10.92) | 1.28  (0.95-1.73) | 1.41  (0.89-2.22) | 1.84  (1.42-2.37) |
|  | Other^[[1]](#footnote-1)^ | NA | NA | NA | 1.96 (0.29-7.53) | 2.63 (0.88-9.62) |
|  | Male | 1 [Reference] | 1 [Reference] | 1 [Reference] | 1 [Reference] | 1 [Reference] |
| Race | |  |  |  |  |  |
|  | Non-white | 0.41  (0.06-1.53) | 0.50  (0.14-1.34) | 0.68  (0.49-0.94) | 0.88  (0.55-1.42) | 0.48  (0.37-0.63) |
|  | White | 1 [Reference] | 1 [Reference] | 1 [Reference] | 1 [Reference] | 1 [Reference] |
| Age range | |  |  |  |  |  |
|  | ≥25 | 2.45  (0.54-8.14) | 0.80  (0.12-2.79) | 0.98  (0.61-1.54) | 1.50  (0.79-2.68) | 0.84  (0.57-1.24) |
|  | 15-24 | 1 [Reference] | 1 [Reference] | 1 [Reference] | 1 [Reference] | 1 [Reference] |
| Education level | |  |  |  |  |  |
|  | Undergraduate | 0.63  (0.19-2.84) | 1.21  (0.41-5.20) | 0.90  (0.61-1.3) | 0.76  (0.44-1.32) | 1.08  (0.77-1.52) |
|  | Graduate | 1 [Reference] | 1 [Reference] | 1 [Reference] | 1 [Reference] | 1 [Reference] |
| Living arrangement | |  |  |  |  |  |
|  | Living in UR | 0.73  (0.22-2.20) | 0.98  (0.41-2.29) | 0.78  (0.58-1.04) | 0.94  (0.61-1.44) | 0.93  (0.73-1.19) |
|  | Not living in UR^[[2]](#footnote-2)^ | 1 [Reference] | 1 [Reference] | 1 [Reference] | 1 [Reference] | 1 [Reference] |
| Work status | |  |  |  |  |  |
|  | Employed | 2.41  (0.80-8.04) | 0.85  (0.34-2.00) | 1.49  (1.11-2.00) | 1.28  (0.84-1.98) | 1.45  (1.12-1.88) |
|  | Non-employed | 1 [Reference] | 1 [Reference] | 1 [Reference] | 1 [Reference] | 1 [Reference] |
| Has in-person class(es) for Fall 2020? (Y/N)^[[3]](#footnote-3)^ | |  |  |  |  |  |
|  | Yes | 0.92  (0.28-4.11 | 5.93  (1.23-106.56) | 1.10  (0.77-1.57) | 1.07  (0.63-1.81) | 1.29  (0.96-1.75) |
|  | No | 1 [Reference] | 1 [Reference] | 1 [Reference] | 1 [Reference] | 1 [Reference] |
| Has medical conditions? (Y/N) | |  |  |  |  |  |
|  | No | 1 [Reference] | 1 [Reference] | 1 [Reference] | 1 [Reference] | 1 [Reference] |
|  | Yes | 2.62  (0.78-7.92) | 4.28 (1.81-10.13) | 2.11  (1.51-2.94) | 4.55  (2.92-7.10) | 2.27  (1.62-3.24) |

1. Odds ratios for this category is indeterminable due to low representation. [↑](#footnote-ref-1)
2. UR: university residences [↑](#footnote-ref-2)
3. Y/N: yes/no [↑](#footnote-ref-3)
