# Supplementary material for: A mixed-methods approach to understand university students’ perceived impact of returning to class during COVID-19 on their mental and general health
Source: PLoS One. 2023 Jan 3;18(1):e0279813. doi: 10.1371/journal.pone.0279813 (PMC9810175; doi:10.1371/journal.pone.0279813)
Supplement: S8 Table — (DOCX) [file pone.0279813.s013.docx]

**Table S8**. Adjusted multivariate regression analysis EQ-5D scales associated with characteristics of survey respondents.

| Characteristic | | Mobility | | Self-care | | Usual activities | | Pain/discomfort | | Anxiety/depression | |
| --- | --- | --- | --- | --- | --- | --- | --- | --- | --- | --- | --- |
|  |  | AOR (95% CI) | *P* value | AOR (95% CI) | *P* value | AOR (95% CI) | *P* value | AOR (95% CI) | *P* value | AOR (95% CI) | *P* value |
| Gender | |  |  |  |  |  |  |  |  |  |  |
|  | Female | 3.08  (0.78-20.47) | .115 | 2.91 (1.04-10.35) | .041* | 1.08  (0.79-1.48) | .639 | 1.21  (0.71-2.11) | .547 | 1.60  (1.23-2.09) | <.001* |
|  | Other | <0.01 | .640 | NA | NA | 3.06  (1.00-9.13) | .0495* | 1.14  (0.16-4.99) | .901 | 2.66  (0.85-10.05) | .094 |
|  | Male | 1 [Reference] |  | 1 [Reference] |  | 1 [Reference] |  | 1 [Reference] |  | 1 [Reference] |  |
| Race | |  |  |  |  |  |  |  |  |  |  |
|  | Non-white | 0.52  (0.08-2.06) | .381 | 0.82  (0.23-2.32) | .730 | 0.72  (0.50-1.01) | .060 | 0.84  (0.44-1.54) | .554 | 0.52  (0.39-0.70) | <.001* |
|  | White | 1 [Reference] |  | 1 [Reference] |  | 1 [Reference] |  | 1 [Reference] |  | 1 [Reference] |  |
| Age range | |  |  |  |  |  |  |  |  |  |  |
|  | ≥ 25 | 5.62  (0.40-80.34) | .210 | 2.28  (0.22-24.72) | .480 | 0.75  (0.37-1.52) | .430 | 3.67  (1.06-13.22) | .325 | 0.84  (0.44-1.57) | .580 |
|  | 15-24 | 1 [Reference] |  | 1 [Reference] |  | 1 [Reference] |  | 1 [Reference] |  | 1 [Reference] |  |
| Work status | |  |  |  |  |  |  |  |  |  |  |
|  | Employed | 1.74  (0.50-6.38) | .380 | 0.59  (0.20-1.55) | .293 | 1.40  (1.01-1.94) | .042* | 1.02  (0.58-1.78) | .822 | 1.36  (1.01-1.83) | .038* |
|  | Unemployed | 1 [Reference] |  | 1 [Reference] |  | 1 [Reference] |  | 1 [Reference] |  | 1 [Reference] |  |
| Living arrangement | |  |  |  |  |  |  |  |  |  |  |
|  | Living in UR | 0.94  (0.25-3.45) | .923 | 0.72  (0.28-1.87) | .495 | 0.79  (0.57-1.09) | .159 | 1.07 (0.62-1.87) | .930 | 0.89  (0.66-1.19) | .425 |
|  | Not Living in UR^[[1]](#footnote-1)^ | 1 [Reference] |  | 1 [Reference] |  | 1 [Reference] |  | 1 [Reference] |  | 1 [Reference] |  |
| Education level | |  |  |  |  |  |  |  |  |  |  |
|  | Undergraduate | 3.18  (0.29-49.57) | .386 | 1.28  (0.24-12.66) | .799 | 1.04  (0.58-1.92) | .889 | 1.59  (0.56-5.33) | .582 | 1.19  (0.67-2.07) | .551 |
|  | Graduate | 1 [Reference] |  | 1 [Reference] |  | 1 [Reference] |  | 1 [Reference] |  | 1 [Reference] |  |
| Has in-person classes for Fall 2020? (Y/N)^[[2]](#footnote-2)^ | |  |  |  |  |  |  |  |  |  |  |
|  | Yes | 1.18  (0.28-6.55) | .832 | 7.77  (1.33-150.73) | .019* | 0.99  (0.66-1.50) | .973 | 1.07  (0.62-1.87) | .705 | 1.03  (0.72-1.46) | .878 |
|  | No | 1 [Reference] |  | 1 [Reference] |  | 1 [Reference] |  | 1 [Reference] |  | 1 [Reference] |  |
| Has medical conditions? (Y/N) | |  |  |  |  |  |  |  |  |  |  |
|  | No | 1 [Reference] |  | 1 [Reference] |  | 1 [Reference] |  | 1 [Reference] |  | 1 [Reference] |  |
|  | Yes | 2.01  (0.58-6.37) | .255 | 1.34  (0.46-2.23) | .003* | 1.89  (1.34-2.68) | <.001* | 4.54  (2.72 -7.57) | <.001 | 1.86  (1.31-2.69) | <.001* |

1. UR: university residences [↑](#footnote-ref-1)
2. Y/N: yes/no

   * significant at ≤0.05 [↑](#footnote-ref-2)
