# Supplementary material for: A mixed-methods approach to understand university students’ perceived impact of returning to class during COVID-19 on their mental and general health
Source: PLoS One. 2023 Jan 3;18(1):e0279813. doi: 10.1371/journal.pone.0279813 (PMC9810175; doi:10.1371/journal.pone.0279813)
Supplement: S9 Table — (DOCX) [file pone.0279813.s014.docx]

**Table S9.** Multiple linear regression analysis of factors associated with overall health score (EQ-5D-VAS) in survey respondents.

| Characteristic | | Beta not adjusted (not standardized coefficient of regression) | P value | Beta adjusted (Standardized Regression Coefficient beta) | 95% CI for Beta | P value |
| --- | --- | --- | --- | --- | --- | --- |
| Gender | |  |  |  |  |  |
|  | Female | 3.06 | .537 | 3.78 | -5.87  13.43 | .443 |
|  | Male | 4.40 | .377 | 4.01 | -5.70  13.73 | .418 |
|  | Other | [Reference] |  | 0.10 |  |  |
| Race | |  |  |  |  |  |
|  | Non-white | 3.44 | .007* | 3.02 | 0.43  5.62 | .023* |
|  | White | [Reference] |  | [Reference] |  |  |
| Age range | |  |  |  |  |  |
|  | 15-24 | -1.99 | .291 | -4.36 | -9.90  1.17 | .122 |
|  | ≥ 25 | [Reference] |  |  |  |  |
| Work status | |  |  |  |  |  |
|  | Employed | -0.58 | .628 | -0.48 | -3.05  2.09 | .714 |
|  | Unemployed | [Reference] |  | [Reference] |  |  |
| Living arrangement | |  |  |  |  |  |
|  | Not living in UR | -0.20 | .864 | 0.14 | 2.43  2.71 | .915 |
|  | Living in UR^[[1]](#footnote-1)^ | [Reference] | [Reference] |  |  |  |
| Education level | |  |  |  |  |  |
|  | Undergraduate |  |  |  |  |  |
|  | Graduate | 0.60 | .710 | -0.25 | -5.09  4.58 | .918 |
| Has in-person class(es) for Fall 2020? (Y/N)^[[2]](#footnote-2)^ | |  |  |  |  |  |
|  | Yes |  |  |  |  | .017* |
|  | No | -1.59 | .267 | -3.85 | -6.99  -0.70 |  |
| Has medical conditions? (Y/N) | |  |  |  |  |  |
|  | Yes | -8.30 | <.001* | -7.96 | -10.91  -5.01 | <.001* |
|  | No | [Reference] |  | [Reference] |  |  |

1. UR: university residences [↑](#footnote-ref-1)
2. Y/N: yes/no

   * significant at ≤0.05 [↑](#footnote-ref-2)
