# Supplementary material for: A mixed-methods approach to understand university students’ perceived impact of returning to class during COVID-19 on their mental and general health
Source: PLoS One. 2023 Jan 3;18(1):e0279813. doi: 10.1371/journal.pone.0279813 (PMC9810175; doi:10.1371/journal.pone.0279813)
Supplement: S10 Table — (DOCX) [file pone.0279813.s015.docx]

**Table S10.** Sensitivity analysis of factors associated with mental health and overall health score, restricted to survey respondents attending in-person class only.

**Table S10.1** DASS-21

| Characteristic | | Depression | | Anxiety | | Stress | |
| --- | --- | --- | --- | --- | --- | --- | --- |
|  |  | AOR (95% CI) | *P* value | AOR (95% CI) | *P* value | AOR (95% CI) | *P* value |
| Gender | |  |  |  |  |  |  |
|  | Female | 1.75 (1.29-2.37) | <.001* | 1.92 (1.41-2.63) | <.001* | 1.77 (1.29-2.45) | <.001* |
|  | Other | 11.93 (2.24-220.78) | .019* | 5.06 (1.55-19.67) | .007* | 3.89(1.22-13.72) | .021* |
|  | Male | 1 [Reference] |  | 1 [Reference] |  | 1 [Reference] |  |
| Race | |  |  |  |  |  |  |
|  | Non-white | 0.86 (0.61-1.21) | .390 | 0.71 (0.50-1.01) | .006* | 0.56 (0.38-0.79) | .001* |
|  | White | 1 [Reference] |  | 1 [Reference] |  | 1 [Reference] |  |
| Age range | |  |  |  |  |  |  |
|  | ≥ 25 | 0.70 (0.29-1.64) | .417 | 1.61 (0.69-3.75) | .263 | 0.60 (0.25-1.40) | .240 |
|  | 15-24 | 1 [Reference] |  | 1 [Reference] |  | 1 [Reference] |  |
| Work status | |  |  |  |  |  |  |
|  | Employed | 1.02 (0.73-1.42) | .896 | 1.28(0.92-1.78) | .136 | 1.19 (0.86-1.68) | .293 |
|  | Unemployed | 1 [Reference] |  | 1 [Reference] |  | 1 [Reference] |  |
| Living arrangement | |  |  |  |  |  |  |
|  | Living in UR^[[1]](#footnote-1)^ | 0.79 (0.57-1.09) | .153 | 0.77 (0.56-1.06) | .103 | 0.61 (0.44-0.85) | .003* |
|  | Not Living in UR | 1 [Reference] |  | 1 [Reference] |  | 1 [Reference] |  |
| Education level | |  |  |  |  |  |  |
|  | Undergraduate | 1.17 (0.59-2.24) | .642 | 1.78 (0.93-3.47) | .079 | 0.99 (0.51-1.90) | .972 |
|  | Graduate | 1 [Reference] |  | 1 [Reference] |  | 1 [Reference] |  |
| Has medical conditions? (Y/N) | |  |  |  |  |  |  |
|  | No | 1 [Reference] |  | 1 [Reference] |  | 1 [Reference] |  |
|  | Yes | 1.88 (1.27-2.82) | .001* | 2.63 (0.82-3.83) | <.001* | 2.49 (1.70-3.68) | <.001* |

**Table S10.2.** EQ-5D

| Characteristic | | Mobility | | Self-care | | Usual activities | | Pain/discomfort | | Anxiety/depression | |
| --- | --- | --- | --- | --- | --- | --- | --- | --- | --- | --- | --- |
|  |  | OR (95% CI) | *P* value | OR (95% CI) | *P* value | OR (95% CI) | *P* value | OR (95% CI) | *P* value | OR (95% CI) | *P* value |
| Gender | |  |  |  |  |  |  |  |  |  |  |
|  | Female | 5.75  (1.02-108.12) | .046* | 2.85  (1.00-10.21) | .048* | 1.14  (0.79-1.63) | .480 | 1.21  (0.71-2.11) | .484 | 1.77  (1.31-2.40) | <.001* |
|  | Other^[[2]](#footnote-2)^ | NA | NA | NA | NA | 2.87 (0.94-8.58) | .058 | 1.14 (0.16-4.99) | .880 | 2.81 (0.88-10.77) | .082 |
|  | Male | 1 [Reference] |  | 1 [Reference] |  | 1 [Reference] |  | 1 [Reference] |  | 1 [Reference] |  |
| Race | |  |  |  |  |  |  |  |  |  |  |
|  | Non-white | 0.35  (0.02-2.00) | .273 | 0.90  (0.25-2.56) | .855 | 0.88  (0.58-1.31) | .533 | 0.84  (0.44-1.54) | .587 | 0.54  (0.38-0.76) | <.001* |
|  | White | 1 [Reference] |  | 1 [Reference] |  | 1 [Reference] |  | 1 [Reference] |  | 1 [Reference] |  |
| Age range | |  |  |  |  |  |  |  |  |  |  |
|  | ≥25 | 13.26  (0.66-352.83) | .093 | 2.91  (0.29-28.92) | .347 | 0.91  (0.37-2.18) | .830 | 3.67  (1.06-13.22) | .041* | 0.71  (0.31-1.64) | .428 |
|  | 15-24 | 1 [Reference] |  | 1 [Reference] |  | 1 [Reference] |  | 1 [Reference] |  | 1 [Reference] |  |
| Education level | |  |  |  |  |  |  |  |  |  |  |
|  | Undergraduate | 2.51  (0.16-71.88 | .560 | 1.27  (0.24-11.68) | .803 | 1.06  (0.55-2.13) | .859 | 1.59  (0.56-5.33) | .403 | 1.29  (0.67-2.50) | .443 |
|  | Graduate | 1 [Reference] |  | 1 [Reference] |  | 1 [Reference] |  | 1 [Reference] |  | 1 [Reference] |  |
| Living arrangement | |  |  |  |  |  |  |  |  |  |  |
|  | Living in UR | 1.46  (0.34-7.09) | .611 | 0.62  (0.23-1.64) | .330 | 0.73  (0.52-1.05) | .088 | 1.07  (0.62-1.87) | .809 | 0.84  (0.61-1.17) | .320 |
|  | Not living in UR^[[3]](#footnote-3)^ | 1 [Reference] |  | 1 [Reference] |  | 1 [Reference] |  | 1 [Reference] |  | 1 [Reference] |  |
| Work status | |  |  |  |  |  |  |  |  |  |  |
|  | Employed | 1.66  (0.38-7.36) | .486 | 0.61  (0.21-1.63) | .328 | 1.38  (0.96-1.98) | .082 | 1.02  (0.58-1.78) | .937 | 1.34  (0.96-1.89) | .082 |
|  | Non-employed | 1 [Reference] |  | 1 [Reference] |  | 1 [Reference] |  | 1 [Reference] |  | 1 [Reference] |  |
| Has medical conditions? (Y/N) | |  |  |  |  |  |  |  |  |  |  |
|  | Yes | 2.32  (0.57-8.50) | .224 | 3.51 (1.41-8.62) | .008* | 1.92  (1.30-2.80) | <.001* | 4.54  (2.72-7.57) | <.001* | 2.31  (1.54-3.53) | <.001* |
|  | No | 1 [Reference] |  | 1 [Reference] |  | 1 [Reference] |  | 1 [Reference] |  | 1 [Reference] |  |

**Table S10.3** EQ-VAS

| Characteristic | | Beta adjusted (Standardized Regression Coefficient beta | 95% CI for beta | P value |
| --- | --- | --- | --- | --- |
| Gender | |  |  |  |
|  | Female | 3.47 | -6.20  13.14 | 0.482 |
|  | Male | 3.48 | -6.26  13.23 | 0.483 |
|  | Other | [Reference] |  |  |
| Race | |  |  |  |
|  | Non-white | 1.98 | -1.05  5.02 | 0.199 |
|  | White | [Reference] |  |  |
| Age range | |  |  |  |
|  | ≥ 25 |  |  |  |
|  | 15-24 | -3.83 | -11.07  3.41 | 0.299 |
| Work status | |  |  |  |
|  | Employed | -0.87 | -3.75  2.02 | 0.556 |
|  | Unemployed | [Reference] |  |  |
| Living arrangement | |  |  |  |
|  | Living in UR^[[4]](#footnote-4)^ |  |  |  |
|  | Not living in UR | -0.34 | -3.15  2.47 | 0.812 |
| Education level | |  |  |  |
|  | Undergraduate |  |  |  |
|  | Graduate | -0.06 | -5.65  5.53 | 0.984 |
| Has medical conditions? (Y/N) | |  |  |  |
|  | Yes | -7.80 | -11.05  -4.56 | <.001* |
|  | No | [Reference] |  |  |

1. UR: university residences

   * significant at ≤0.05 [↑](#footnote-ref-1)
2. Odds ratios for this category is indeterminable due to low representation. [↑](#footnote-ref-2)
3. UR: university residences

   * significant at ≤0.05 [↑](#footnote-ref-3)
4. UR: university residences

   * Significant at ≤0.05 [↑](#footnote-ref-4)
