# Supplementary material for: A mixed-methods approach to understand university students’ perceived impact of returning to class during COVID-19 on their mental and general health
Source: PLoS One. 2023 Jan 3;18(1):e0279813. doi: 10.1371/journal.pone.0279813 (PMC9810175; doi:10.1371/journal.pone.0279813)
Supplement: S11 Table — (DOCX) [file pone.0279813.s016.docx]

**Table S11.** Key themes in respondents’ feedback from focus group discussions.

| Theme | Participants,  n (%) | Example |
| --- | --- | --- |
| Attending class during COVID-19 is associated with unhealthy behaviors and poor health | 18 (75.0%) | I actually started missing meals and stuff. I’d just sit—sit here at my desk—lecture, homework, project, lecture, homework, project, sometimes I just forget to eat and then I’ve just realized that I started to lose weight significantly. So, I don’t think this is a very healthy lifestyle. (Subject 024)  My lack of being able to manage myself with asynchronous learning um, because of my anxiety just led to more um sedentary, relaxed time and then short bursts of panic and stress, um... and lack of sleep, um, when deadlines hit or like um, when it's time to actually submit everything. So adding more onto procrastination and stress that's kind of uh, out of that has definitely increased. (Subject 006)  …especially in times of stress I find that, like, I lose my appetite a lot and for the past couple of years I’ve been doing really well at balancing, but like since this semester started between like, the anxiety of COVID and the stress of classes and stuff, I have noticed that I’m losing weight again, which is not ideal for me. (Subject 016)  Yeah, I've experienced a lot of that weight gain also. I call it my 'COVID-19' instead of the 'freshman 15'. It's definitely difficult just with a lot of- just the isolation and being alone, working asynchronously on school. So, like, my desk is right in my kitchen, I'm in a small apartment so, I'm sitting here for 12 hours a day doing homework sitting right next to all of my snacky food and refrigerator that I know I have all this food in so... the school aspect of being uh, so much of it online has definitely negatively affected my diet. And the closures overall of just throughout the city, throughout the country have also made it very difficult to stay active. (Subject 002)  I definitely feel like I’m exercising a lot less since I’m not going to campus, I’m not walking between buildings. I don’t really have a reason to go outside since everything can be done indoors and the gym so far. And then it just- makes it really difficult to motivate when you don’t have to move. (Subject 020)  I definitely could um relate to the fact that I have less classes in person so that's less walking to and from- to different classes. Which I think was, honestly, a lot of my physical activity um my first year of college. And then, also my first year at college I spent a lot of time um at the gym whenever I could get the chance to, but I think nowadays with the hassle of having to wear a mask while you exercise and um even just like wiping down equipment um just makes the whole ordeal seem to not be worth it. (Subject 004)  I'm um, a rock climber so I would normally be training for 5 days a week, and just the gym's closed, the wall's closed um... and a lot of other places where I would normally be training were just completely closed so I don't have that physical activity which always helped me stay focused and so now, that's not there, which makes it more difficult in school. Um... so, definitely a lot of secondary negative health impacts because of that just more sedentary lifestyle and isolation. (Subject 002)  Being online it was really easy to just like, “Oh, maybe I’m going to sleep through this one and I’ll watch it later...” things kind of piling up and then the stress that comes with all that was also, not, easy to deal with. (Subject 022)  Additionally, primarily during the initial shutdown I’d just never really left my apartment much so it’s like, whereas previously like going to work you’d at least be up on your feet, walking around, walking to lab, walking in between classes, what have you, for really a month and a half, it might be you move less than a thousand steps in a whole day if you didn’t make an effort to actually go for a walk or go for a run, or go do something outside, and so there defiantly were days where I’d truly become sedentary as opposed to at least getting some kind of physical exertion, whether it be walking up and down stairs, or whatever. (Subject 012) |
| Perceived challenges of online learning and increased feelings of isolation | 17 (70.83%) | I think the thing I’ve experienced a lot as well is a lot of, it’s hard to communicate online. A lot my professors have been somewhat, not intentionally, but vague in assignments or it’s hard to get a hold of people. (Subject 013)  It also just felt kind of trapped. I spent basically my entire days just sitting in my room, watching lectures and then going back to doing problems back and forth, back and forth. It just felt like I was heading into a more and more isolated, like, situation. (Subject 024)  We're spending most of our times online, so when it comes to socializing online with clubs it doesn't sound appealing, which definitely does impact how social you want to be. So, I feel like everyone's been trying to make an effort to make everything online to be accessible, but it's kind of having the opposite effect. (Subject 002)  I’ve realized all my interactions with my colleagues like, in the program where I teach are gone. And um then what that ends up doing psychologically, all the small issues with students or just the difficulties that are kind of endemic to teaching in graduate school- like I don’t have that camaraderie and that social support and routine that usually helps me take care of those things really quickly. (Subject 011)  I also have to feel the stress of okay, my computer doesn't work? Well, we had an internet outage today, and I can't go to campus, how am I goanna tell that to my professor? Like, these extra conditions of the real world impacting the online um learning experience has also been an added stress... an added factor. (Subject 006)  Um I’m teaching this semester, and before when I was in class, I could go in early, and get to know my students a little bit more, but now that I am teaching over zoom predominantly, I feel like I don’t have that contact with them, so I feel much more psychologically distant from them as well as physically distant from them, so that was kind of unfortunate this semester. (Subject 009)  …I realized it’s started really tiring me so much, even though it is a less amount. Like I used to sit in the classes for a longer time, and even teach after like having the classes, but online version starts to tire me more quickly. I don’t know like maybe because of like electronics also the other stuff going on this house. (Subject 008)  I've noticed the college clubs and stuff that I participated in last semester and the semester before that um have obviously all transitioned over to being virtually. And I've noticed for me like it's not really- it's not really worth my time now just to be virtually participating in those clubs so I kind of have stepped away from that and not involved with any of those clubs anymore and just, and basically just focusing on my uh classes here online. (Subject 001)  I think one of the unexpected things that happened was you know I’m teaching virtually, and attending classes virtually, and I didn’t realize how much I would have to see my own face and my body all the time on a camera, and that was kind of an unexpected psychological effect. (Subject 011)  In terms of seeing people, I feel like in a lot of my classes people don’t have the cameras on, so I feel like I’m interacting with people less even though I’m not necessarily communicating less I just feel like I’m around people less often. (Subject 013) |
| Demands for COVID-19 policy reform and greater transparency of university COVID-19 statistics | 17 (70.83%) | I often feel like, I take all the individual actions that they want me to do because that’s the right thing to do, but I don’t have a lot of confidence that the university takes larger measures over the spaces it controls that complement and enhance the individual actions. …my individual actions only go so far in terms of my safety and the safety of others. (Subject 011)  I don’t really understand why they didn’t want to test me because I do go on campus, I also teach a class, so it’s like the amount of people I’m exposed to, I feel like, you should test me because I don’t want to risk anyone else’s health and safety because I was potentially exposed at the time. (Subject 019)  So, right now it’s basically well, we’ll all wear our masks and come back to campus, and that’s just how it’s going to be. But that really doesn’t take into account different levels of spread, and levels of dangers…. Making that plan public, and those gradations really clear would be a major, major right step in our pandemic response, and I think, in making, giving students, faculty, and staff confidence in our university’s ability to care for people on its campus. (Subject 011)  I think there needs to be more of a transparency and a little bit more effort in managing our like, spaces and managing our risk situation… because not only did they not um, count classroom spaces as high-risk places, they don't count any of our offices as high-risk spaces, and they don't count any of our research spaces as high-risk spaces, so there's a lot of things that can slip through those cracks, and a lot of us are really scared and uncomfortable… Within classrooms, and within other enclosed spaces, um, like, they're not doing a really good job of informing people of what risks they might be under, who they might be in contact with, so contact tracing has also been very uninformative and um, not really doing a lot. (Subject 006)  …yeah so, the professor took off the mask to cough and then put it back on afterwards, and just kind of hearing that and picking up little stories, kind of like that it sort of felt like the professors themselves weren’t being held to the same standard that students were, which kind of doesn’t fit right with me because it felt like if anything, they should be held to a higher standard. (Subject 022).  I was going to say I had a similar experience. One of the other people in my lab section, her roommate had tested positive, and she chose to self-isolate for two weeks, and also get a negative test, but she had mentioned that it was odd that Purdue did not require her to self-isolate. They do live off-campus, they cook and eat together, and it was just odd to me how she did not get contact traced, and it was, at least, once again pleasantly surprising that she chose to self-isolate and quarantine herself, but I don’t know how many people are doing the same, in fact it sounds like others are not. So, I think that’s kind of a gray area that should be more enforced and defined. (Subject 001)  So not just in how policies are communicated or talked about or whatever, but really, to be safer here, we need different policies that are much, much more concrete, and are really more geared toward public health and not sort of financial gain or staying open or whatever. (Subject 011) |
| Difficulties in adhering to COVID-19 related policies and protocols implemented by the university. | 13 (54.17%) | Someone I was standing with mentioned that their roommate had recently tested positive for COVID, and then it’s like okay, I think it’s fairly reasonable to say if your roommate, who you’ve been in close contact with for a significant period of time, most likely without a mask on, has tested positive, you probably shouldn’t be out in public, even if you are still socially distanced, and what not. (Subject 012)  I do not wipe down the computer desktop or the keyboard after- after I'm done with recitation or doing a- or hosting a teaching period. Um... out of- one, of course, laziness- I won't deny that, but also my uh, my other TA's who come in after me say it's not a big deal, I know you're not COVID positive, so you don't need to waste your time or my time by wiping down the- the keyboard after the fact. (Subject 007)  I live in … and that’s where all the bars are and I would say once it hits Thursday night, the social distancing and all of that kind of goes out the window… it is concerning cause it’s like, these are all students and even if they do adhere to all the guidelines on campus, like this is what they’re doing on their weekends, so like I don’t really know how that would play out, like, are they really protecting themselves or is it just like when they’re on campus. (Subject 019)  I know there’s a lot of shame associated with, like, having it and not saying anything, so like, one time one of my friends thought that they had it and they were like, “Oh, I don’t know if I’m going to tested, like that’s so embarrassing," and I was like, “Well, but it’s not safe for you to not get tested and not tell anyone.” So then they ended up getting tested so like, I definitely think people are more nervous to admit that they might have it just because even if they’re doing everything to protect themselves and the people around them...but...um...yeah. I think it just going along with like the standard to, um, admit that you have it and get tested. (Subject 017)  We all should be wearing a mask and wearing it correctly, like especially now like see I'll just go walking outside. People hang out wearing masks, but not wearing it correctly. Like they don't have it covered over your nose…  (Subject 005)  …or just like there was a photo on Reddit of president of the school not wearing a face mask in public which really felt discouraging for a lot of students because when we see that the head of the university isn’t doing the thing they’re kind of preaching, it feels like the guidelines are just there to prevent law suits, than to actually make a change. (Subject 015)  …as students working a lot of time and often eating lunch and working at the same time, it's been very difficult to like, find places to eat or have video calls on campus while adhering to the policies and not being in a room full of people all having video calls if you're off campus and you don't have time to go home. (Subject 006)  So I used to work for dining and catering so I was helping out during orientation, and that was something else I noticed that you know some of the TL’s were doing their best to enforce you know, six feet apart in the lines, and they were trying to, you know, break up the groups of five or six friends that were standing together, but people weren’t really listening, people were wearing their masks, they were either not wearing them or wearing them below their nose. (Subject 014)  There’s a couple of instances where I think that the policies haven’t necessarily been adhered to as much as I would expect or not necessary as much as I would expect, but some instances where I felt less safe was when I noticed that in the building where my office is, they have people that come through and spray the door handles, and like wipe down the door handles periodically, and they’re — I understand it’s a very thankless job, and just a very tedious job, but they would just like spray the door handle, wouldn’t really wipe them down, and so like now you have half the door handle is just wet, and the other half, the back side where people are actually grabbing, hasn’t been wiped off or anything with any disinfectant, so it’s just like okay, is this actually doing anything or is now my hand just wet for no purpose? (Subject 022) |
| Concerns about acquiring COVID-19 and transmitting it to close contacts | 8 (33.33%) | I don’t really understand why they didn’t want to test me because I do go on campus, I also teach a class, so it’s like the amount of people I’m exposed to, I feel like, you should test me because I don’t want to risk anyone else’s health and safety because I was potentially exposed at the time. (Subject 019)    … I kind of wanted to add to the frustration about getting tested, like one of my family members is immunocompromised, um, and I’m not going home for Thanksgiving, I’m only going home until December, but as I understand that there’s departure testing for Thanksgiving, but after that it feels like they’re kind of stopping testing, which doesn’t make sense for the people who didn’t go home at Thanksgiving but kind of expect to go home in December, and- so it feels like I just kind of have to get tested and then just coup up more- kind of more strictly in my apartment. (Subject 022)  … but being immunocompromised, it’s very important to me that people, you know, adhere to the guidelines when possible because I — this is not a disease that I would like to contract or even take home to my parents. (Subject 013)  So, I don't think we can really come to a conclusion with the severity that the students have had considering there are so many other factors at play. So that does not make me feel any safe, that actually makes me feel more uncomfortable because that just means the student body is now not taking it seriously because they think it's going to pass like a common flu, and they might put me, my boss, any other teaching staff, any of my colleagues at more risk. (Subject 002) |
